# Advances in engineered T cell immunotherapy for autoimmune and other non-oncological diseases

**DOI:** 10.1186/s40364-025-00736-8

**Published:** 2025-02-04

**Authors:** Qiaolin Huang, Xiaojian Zhu, Yicheng Zhang

**Affiliations:** 1 Department of Hematology, Tongji Hospital, Tongji Medical College, Huazhong University of Science and Technology, 1095 Jiefang Avenue, Wuhan, 430030 Hubei China; 2 Key Laboratory of Organ Transplantation, NHC Key Laboratory of Organ Transplantation, Key Laboratory of Organ Transplantation, Ministry of Education, Chinese Academy of Medical Sciences, Wuhan, 430030 Hubei China; 3 Immunotherapy Research Center for Hematologic Diseases of Hubei Province, Wuhan, 430030 Hubei China

**Keywords:** Engineering T cells, CAR T cell therapy, Autoimmune diseases, Fibrosis, Aging

## Abstract

Adoptive immunotherapy using engineered T cells expressing chimeric antigen receptors has shown remarkable success in treating patients with hematological malignancies. However, realizing broader therapeutic applications of engineered T cells in other diseases requires further exploration in clinical investigations. In this review, we highlight recent advances in the engineering of T cells in non-oncology areas, including autoimmune and inflammatory diseases, infections, fibrosis, hemophilia, and aging. Chimeric antigen receptor immunotherapy has shown good outcomes in non-oncology areas, but many challenges remain in improving its safety and efficacy and and expanding its application to the treatment of non-oncological diseases.

## Background

Chimeric antigen receptor (CAR) immunotherapy, an adoptive cell transfer therapy, has gained popularity in recent decades. CARs exhibit an exceptional capacity to detect surface molecules without requiring presentation or processing by major histocompatibility complex (MHC) molecules, thereby binding to tumor-specific antigens on the surface of malignant tumor cells upon specific recognition. Currently, CAR T cell therapy is mainly used to treat tumors, particularly hematological diseases, such as lymphoma, leukemia, myeloma, and some solid tumors [1]. However, many studies have targeted CAR immunotherapy for non-oncological diseases, such as autoimmune diseases, infections, fibrosis, aging, and graft-versus-host disease (GvHD), with results suggesting that it is feasible to exploit the specificity of CAR immunotherapy to treat these diseases. This review is based on a comprehensive search of relevant studies from PubMed and Web of Science, with primary keywords including ‘chimeric antigen receptor,’ ‘non-oncological diseases,’ and ‘autoimmune diseases,’ among others. This review explores the clinical applications of CAR immunotherapy in non-oncological diseases, with a focus on CAR T cells, CAR-regulatory T cells (Tregs), and relevant experimental and preclinical findings.

## CAR structures

**Fig. 1:**
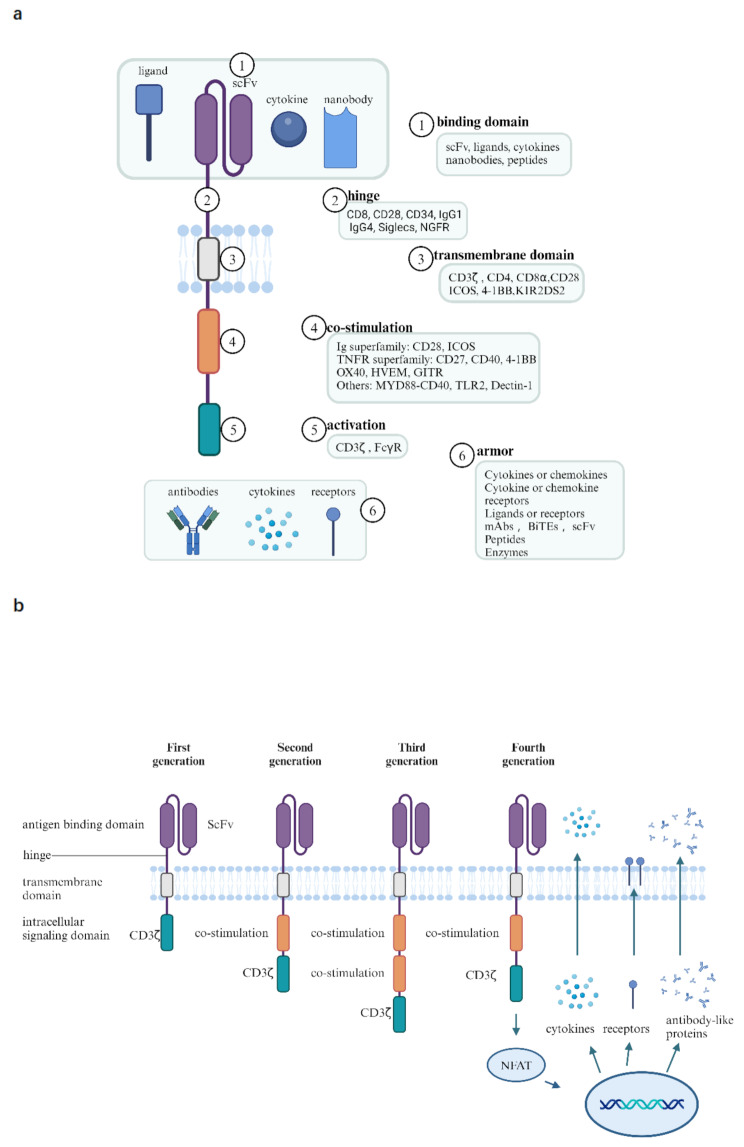
Descriptions of chimeric antigen receptor (CAR). **(a)** Variations in different domains of CAR. **(b)** Development of chimeric antigen receptor (CAR) T cells. In the first-generation CARs, CD3ζ domain was added. In the second-generation CARs, a co-stimulatory domain, 4-1BB or CD28, was added along with the CD3ζ domain. The third generation CARs include two co-stimulatory domains, usually 4-1BB and CD28. The fourth generation CARs include the inductive activation of recombinant immune regulators also called armor, such as IL-12 expression system in addition to the third generation CAR components

**Fig. 2:**
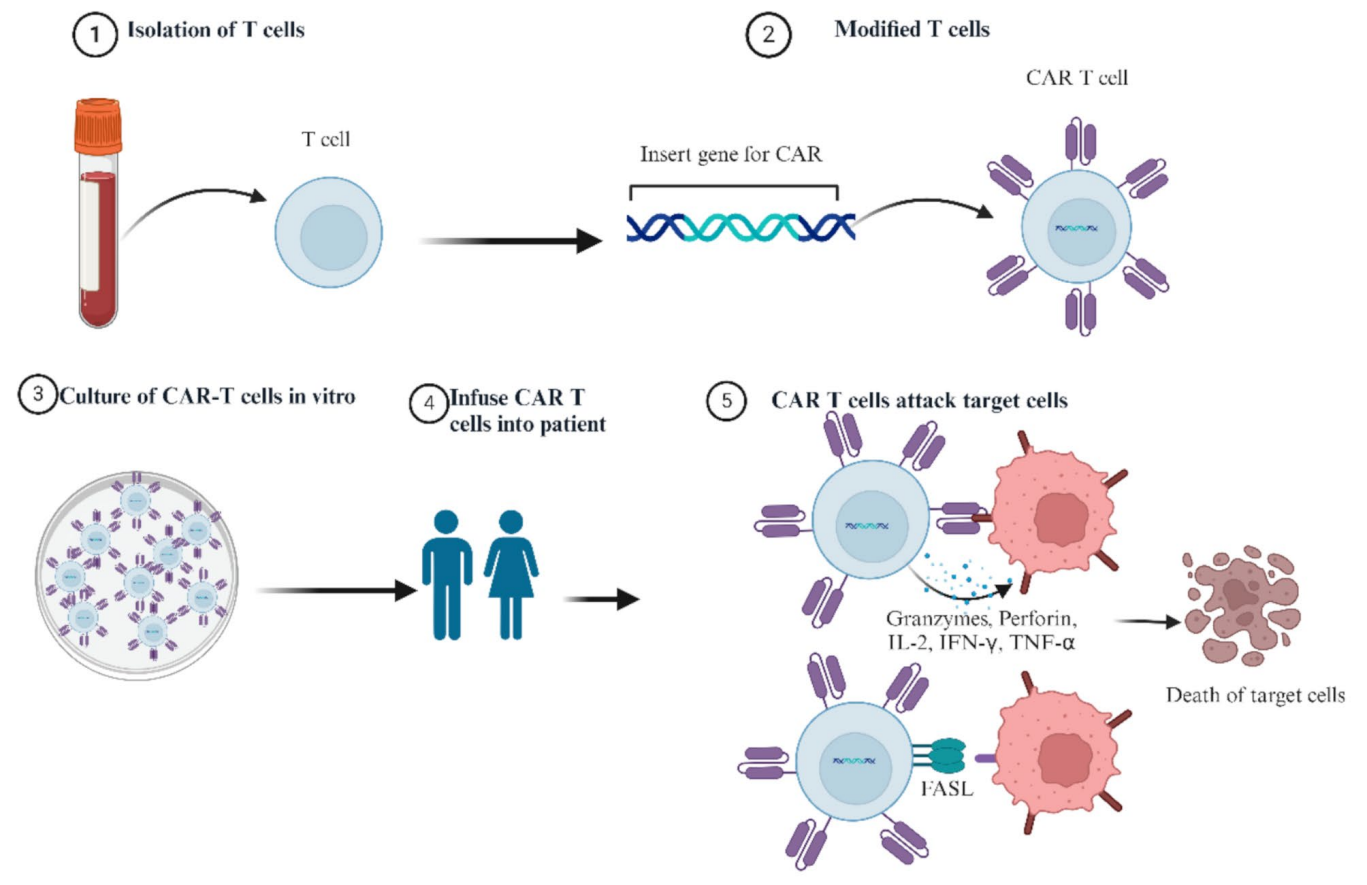
Steps in chimeric antigen receptor T (CAR T) cell therapy: (1) Isolation of T cells from the peripheral blood of patients. (2) Modified T cells: CAR genes are transferred into T cells. (3) Culture of CAR T cells in vitro. (4) Infusion of CAR T cells back into the patients. (5) CAR T cells target and lyse target cells via three axes [198]: [1] Perforin and granzyme [2]. Cytokine secretion, such as IFN-γ and TNF-α [3]. Fas and FasL

CAR comprises three fundamental components: an extracellular domain including an antigen-binding domain and a hinge region, a transmembrane domain, and an intracellular signaling domain [2–4] (Fig. 1a and b). Extracellular target antigen-binding domains, such as single-chain variable fragments (scFvs), ligands, cytokines, nanobodies, and peptides, are primarily responsible for recognizing and binding target antigens [5]. The intracellular signaling domains consist of co-stimulatory factors (e.g., CD27, CD28, 4-1BB, OX40, ICOS, MYD88-CD40, KIR2DS2) and the activation domain CD3ζ. Bispecific CAR, tandem CAR, inhibitory CAR, physiological CAR, universal CAR, and clustered regularly interspaced short palindromic repeats (CRIPSPR) gene editing CAR have also been developed [3, 6–8]. According to the different cells, they are classified as CAR T [9], CAR natural killer (NK) [10], CAR macrophage [11], CAR Treg [12], CAR γδT [13], and CAR dendritic cells (NCT05631899). CAR T cells can specifically identify tumor cells in the body and release several effectors to efficiently kill tumor cells to treat malignant tumors [2], and the CAR T production process and killing mechanisms are shown in Fig. 2. CAR NK cells have a better cell-killing effect than CAR T, but the lifespan of NK cells is short and the suppressive tumor microenvironment may limit their efficacy [14, 15]. CAR macrophages can target and phagocytose tumor cells, alter the tumor microenvironment, and present tumor antigens to T cells [16, 17]. Currently, CAR macrophages face challenges such as limited macrophage proliferation, cytotoxicity, and weak antitumor ability [17]. Tregs are CD4 + CD25^highCD127^lowFOXP3 + cells with context-dependent suppressive activity. Therapeutically, CAR Tregs attenuate autoimmune diseases (e.g., type 1 diabetes mellitus, alloimmune graft-versus-host disease, and transplant rejection) [18, 19]. However, the performance and stability of CAR Treg in inflammatory environments remain to be validated [19].

CAR T cells are evolving rapidly and have reached their fifth generation [2, 5, 20] (Fig. 1b). The first-generation CARs only contain the CD3ζ signaling domain [6], which leads to poor signaling, limited proliferation, and short in vivo survival, resulting in ineffective anti-tumor effects [2]. In second-generation CARs, a co-stimulation domain such as 4-1BB or CD28 is added to improve T cell activation and response, showing better therapeutic outcomes. The third-generation CARs include both 4-1BB and CD28 co-stimulatory domains, aiming for stronger activation, but studies show they do not outperform second-generation CARs [21–24]. The effectiveness of co-stimulatory domains is still debated. The fourth-generation CARs further modify the expression of immune regulators, such as the IL-12 expression system, to enhance T cell function or favorably modify the tumor microenvironment [25]. The fifth-generation CAR T cells, also called universal CAR T cells, refer to T cells made by obtaining T cells from healthy volunteers. Their main advantage is that they can be made available off-the-shelf instead of obtaining T cells from the patients for customization, thus saving time and treatment costs. However, allogeneic T cells can cause GvHD and these cells may be rapidly eliminated by the host immune system, limiting their anti-tumor activity [26]. The basic structure of the current CARs does not change significantly and is usually derived from the original.

## Applications

**Fig. 3:**
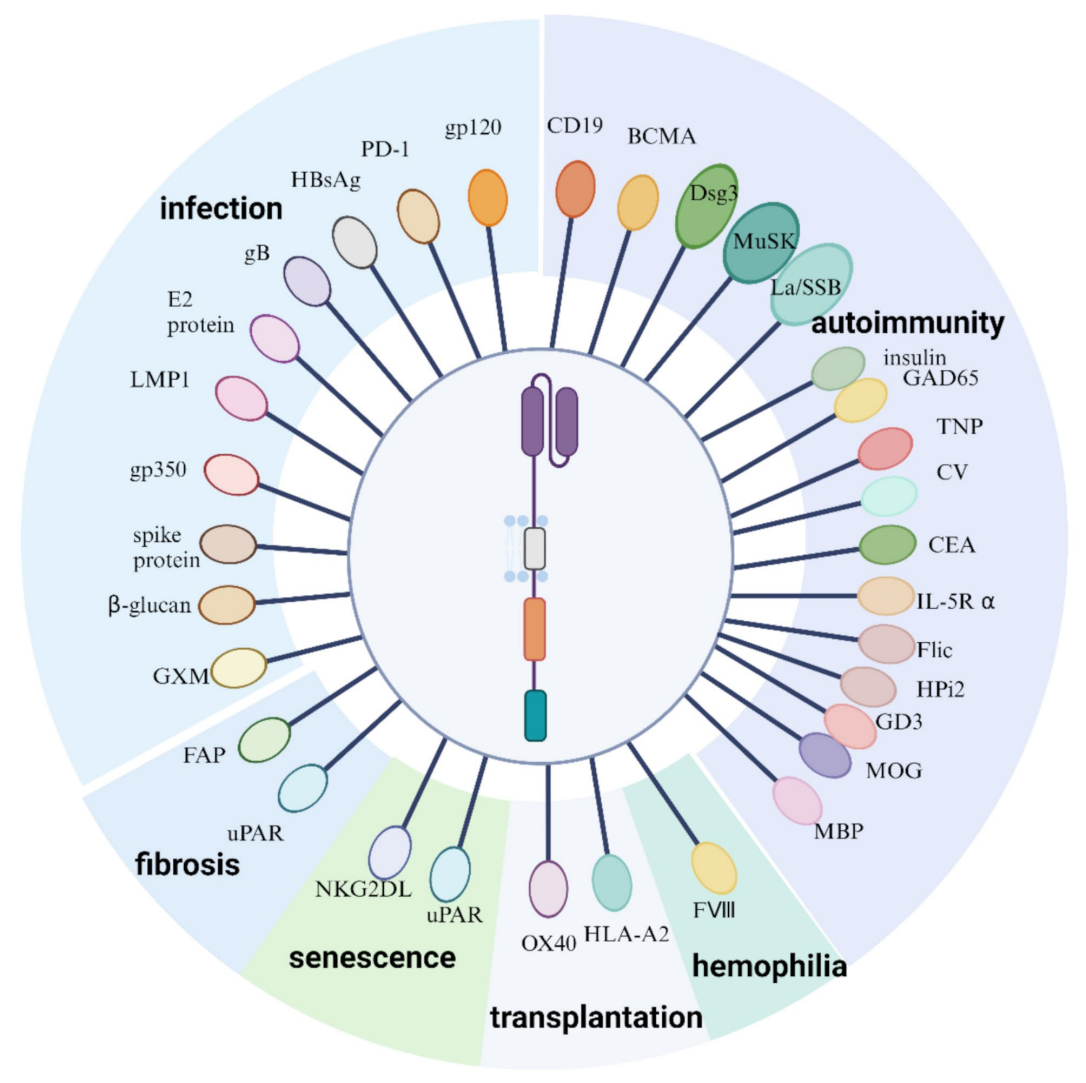
Applications and different targets of CAR immunotherapy in the non-oncology field

In non-oncology, CAR immunotherapy has applications in autoimmune and inflammatory diseases, infections, fibrotic diseases, aging, and more. In the following section, we introduce the applications of CAR immunotherapy in these fields (Fig. 3). Antigen targets and clinical trials with reported data for non-oncological diseases are presented in Tables 1 and 2.

**Table 1:** Targets in non-oncological diseases

Disease	treatment	Target	Structural domain	Animal model	Outcome	Reference
RA	CAR T	citrullinated autoantigens-specific B cell	CD3ζ, 4-1BB	CIA	redirection and killing of self-reactive B cell subpopulations	[50]
	CAR Treg	CV	-	-	-	[53]
SS	CAAR NK	anti-La/SSB B cell	CD3ζ, 4-1BB, CD28	-	elimination of the La/SSB autoreactive B cells	[58]
MS	CAR Treg	MBP/MOG	CD3ζ, CD28	C57Bl/6	suppressing EAE symptom	[68]
MG	CAAR T	anti-MuSK B cell	CD3ζ, 4-1BB	NSG	reduction of the anti-MuSK IgG	[73]
PV	CAAR T	anti-Dsg3 B cell	CD3ζ, 4-1BB	NSG	clinical and histologic resolution of blisters	[88]
T1D	CAR T	MHC class II	CD3ζ, CD28, 4-1BB /CD3ζ, CD28	NOD	delay in the development of T1D.	[98]
	CAR Treg	insulin	CD3ζ, CD28	BALB/cJ, C57BL/6J and nonobese diabetic mice	suppression of diabetes	[99]
IBD	CAR Treg	TNP	-	TNP-specific transgenic mice	amelioration of colitis	[103]
	CAR Treg	CEA	CD3ζ, CD28	CEABAC-2 and CEABAC-10	improvement in ulcerative colitis and prevention of colorectal cancer	[104]
	CAR Treg	FliC	CD3ζ, CD28	NSG	promotion of the establishment of colon-derived epithelial cell monolayers	[105]
asthma	CAR Treg	CEA	CD3ζ, CD28	CEA transgenic C57BL/6	reduction of airway hyperresponsive inflammation and prevention of excess lung mucus	[109]
	CAR T	IL-5 receptor α	CD3ζ, CD28	B6, NSG, Cas9 transgenic mice	repression of lung inflammation and alleviation of asthmatic symptoms	[197]
HIV/SIV	CAR T	gp120	CD3ζ	-	no significant alteration in viral reservoir	[115, 116]
	CAR T	HIV envelope glycoprotein	CD3ζ, 4-1BB	-	inhibition of replicating viruses and specific killing of HIV-infected cells.	[121]
	CAR T	PD-1	CD3ζ, 4-1BB	rhesus macaques	PD-1 + T cell exhaustion	[129]
HBV	CAR T	HBsAg	CD3ζ, CD28	C57BL/6, HBVtg HBV1.3xfs mice, FRG	recognizing soluble HBsAg and eliminating HBsAg-positive hepatocytes	[135–137]
HCV	CAR T	HCV/E2 glycoprotein	CD3ζ, CD28	-	lysis of HCV-infected hepatocytes	[140]
CMV	CAR T	gB	CD3ζ, CD28/4-1BB	NRG	control of HCMV infection	[143]
EBV	CAR T	gp350	CD3ζ, CD28	NRG	reduction of EBV spread and lower frequencies of EBER B cell malignant lymphoproliferation	[148]
COVID-19	CAR NK	envelope spike protein	CD3 ζ, 4-1BB	-	ability to specifically kill pseudo-SARS-CoV-2 infected target cells	[151, 152]
*Aspergillus fumigatus*	CAR T	cell wall	CD3ζ, CD28	NSG	ability to confer antifungal reactivity in preclinical models in vitro and in vivo	[153]
	CAR T	β-glucan	CD3ζ, CD28	NSG	injury and inhibition of *Aspergillus* filament growth in vitro and in vivo	[155]
*Cryptococcus neoformans*	CAR T	glucuronoxylomannan	CD3ζ, CD28	NSG	the control of cryptococcosis	[157]
cardiac fibrosis	CAR T	FAP	CD3ζ, CD28	C57BL/6, *Periostin*^*MCM*^ mice, *RosaOVA* mice, OT-I mice	reduction in cardiac fibrosis	[162]
hepatic fibrosis	CAR T	uPAR	CD3ζ, CD28	C57BL/6 N, NSG	reduction in liver fibrosis	[167]
hemophilia	CAR T	anti-FVIII B cell	CD3ζ, CD28	C57BL/6	capable of killing FVIII-reactive B-cell hybridomas	[174]
	CAR Treg	FVIII	CD3ζ, CD28	E16xDR	ability to suppress the proliferation of FVIII-specific T-effector	[175]
senescence	CAR T	uPAR	CD3ζ, CD28	C57BL/6 N, NSG	ablation of senescent cells	[167]
	CAR T	NKG2D ligand	CD3ζ, 4-1BB	C57BL/6	ability to kill senescent cells in both aged mice and aged nonhuman primates	[183]

Notes: RA, rheumatoid arthritis; SS, Sjögren’s syndrome; CV, citrullinated vimentin; MS, multiple sclerosis; EAE: encephalomyelitis; MuSK, muscle-specific tyrosine kinase; TNP, 2,4,6-trinitrophenol; MG, myasthenia gravis; PV, pemphigus vulgaris; T1D, type 1 diabetes; IBD, inflammatory bowel diseases; MBP, myelin basic protein; MOG, myelin oligodendrocyte glycoprotein; Dsg, desmoglein; CEA, carcinoembryonic antigen; FliC, flagellin derived from Escherichia coli H18; uPAR, urokinase-type plasminogen activator receptor; gB, glycoprotein B; gp350, envelope glycoprotein 350

**Table 2:** Clinical trials with reported data in non-oncological diseases

Disease	Treatment	Types	No.	Patients’ characteristics	Outcomes	Reference
SLE	anti-CD19 CAR T	Case report	1	A 20-year-old woman with severe and refractory SLE	no adverse events; SELENA 16 − 0; clinical and serological improvement	[41]
	anti-CD19 CAR T	Trial	5	5 patients (4 women,1 man) with SLE refractory to several immunosuppressive drug treatments	grade-1 CRS (60%); SLEDAI (8–16)-0; clinical and serological improvement, no relapses during up to 17 months of follow-up, drug-free remission.	[42]
	anti-CD19 CAR T	Case report	1	A 32-year-old woman with SLE during pregnancy at week 33 of gestation	no adverse events; SELENA 10 − 0; clinical and serological improvement	[43]
	anti-CD19/BCMA CAR T	Case report	1	41-year-old woman with SLE and DLBCL	decrease of ANA, increase of C3, and C4; SLE stable and DLBCL in remission, no relapses during up to 17 months of follow-up, drug-free remission.	[45]
	anti-CD19/BCMA CAR T	Trial	12	-	grade-1 CRS (100%), grade-4 hematologic toxicity (91.7%), and grade-3 hematologic toxicity (8.3%); SLEDAI 18.3–1.5; no relapses during up to 6 months of follow-up, drug-free remission.	[44]
	anti-CD19/BCMA CAR T	Trial (phase 1, NCT04162353, NCT05474885)	13	10 women and 3 men; 2 SLE patients combined with DLBCL; median age of 31 years	grade I CRS, neutropenia; 2 SLE patients with DLBCL, SLEDAI (4,8)-0, symptom-free and drug-free remission for 46 and 25 months respectively. remaining 11 patients, average SLEDAI 10.6–2.7, symptom reduction and drug-free remission within 3 months	[46]
SS	anti-CD19 CAR T	Case report	1	a 76-year-old woman SS and DLBCL	grade-2 CRS and grade-1 neurotoxicity; ESSDAI 5 − 2; clinical and serological improvement	[59]
MS	anti-CD19 CAR T	Case report	2	a 47-year-old woman with a 23-year history of MS a 36-year-old man with a 5-year history of MS	woman, grade-1 CRS man, no adverse events	[67]
MG	anti-CD19 CAR T	Case report	1	a 33-year-old woman with a 11-year history of MG	grade-1 transaminitis, 70% reduction in anti-AchR antibodies; clinical improvement	[74]
	anti-BCMA CAR T	Trial (phase 1b/2a, NCT04146051)	14	10 women and 4 men; age 18–83; diagnosis of MG with presence of an MG-associated autoantibody	1 patient with grade 3 urticaria; 2 participants with IVIg dependence and 3 participants with induced MSE; clinical improvement	[75]
	anti-BCMA CAR T	Case report	2	a 33-year-old woman and a 60-year-old woman	cytopenia; clinical improvement	[76]
NMOSD	anti-BCMA CAR T	Trial (phase 1, NCT04561557)	12	10 women and 2 men; median age of 49.5 years	all patients with grade-1/2 CRS; 11 (92%) patients with a drug-free remission and no relapse; all patients with clinical improvement	[78]
ASS	anti-CD19 CAR T	Case report	1	A 41-year-old male patient was ASS	grade-1 CRS clinical and serological remission	[81]
	anti-CD19 CAR T	Case report	1	A 41-year-old man with refractory ASS	grade-1 CRS clinical and serological remission	[82]
	anti-CD19 CAR T	Case report	1	A 44-year-old Caucasian woman with ASS	grade-1 CRS, potential grade-1ICANS clinical and serological remission	[83]
HIV	CD4 CAR T	Trial (phase II)	24	over the age of 13 years on stable antiretroviral therapy for more than 8 weeks with viral loads of 1000 to 100 000 copies/mL and CD4 counts greater than 50/ µL	no adverse events; decrease in HIV RNA levels in rectal tissues, but no significant change in plasma HIV RNA	[115]
	CD4 CAR T	Trial (phase II)	40	20 receive CD4 CAR T, plasma viral loads <50 copies/ml.	no serious product-related adverse events; decrease in HIV burden and suppression in virologic rebound	[116]
	anti-HIV-1 CAR T	Trials (phase 1, ChiCTR2000028826)	18	14 men, and 4 women; median age was 31 years; plasma HIV-1 RNA < 20 copies/mL before enrollment and had no comorbidities or complications.	reduction in viral load (67.1%) and suppression in virologic rebound (74.3%), sustained reduction of HIV-1 RNA levels in 10 patients over the 150-day observation period	[125]
	bNAb-derived CAR T	Trial (phase 1, NCT03240328)	15	15 men, the median CD4 + T cell count of 597 cells/µL	no adverse events reduction in viral reservoir and suppression in the viral rebound	[126]

Notes: SLE, systemic lupus erythematosus; SS, Sjögren’s syndrome; ASS, antisynthetase syndrome; MS, multiple sclerosis; MG, myasthenia gravis; NMOSD, neuromyelitis optica spectrum disorder; DLBCL, diffuse large B cell lymphoma; SELENA, Safety of Estrogens in Lupus National Assessment;ESSDAI, EULAR Sjögren’s Syndrome Disease Activity Index

### Autoimmune and inflammatory diseases

The breakdown of immune tolerance is the basis of pathological autoimmune diseases [27]. The development of autoimmune diseases involves a combination of genetic and environmental factors, leading to the formation of self-reactive T cells, B cells, and autoantibodies, which damage organs and tissues [28]. Therefore, it is crucial to control abnormal T and B cell functions. Currently, glucocorticosteroids and immunosuppressants are used to manage autoimmune diseases, but some patients do not respond optimally [29]. Biological drugs, such as monoclonal antibodies like rituximab, target B cells and offer new treatment options. However, due to the inability to completely remove circulating and memory B cells, some patients still experience poor efficacy and relapse after discontinuing these drugs [30]. CAR T cell therapy, which can be directed to target cells and proliferate extensively to exert cytotoxic effects, is currently approved and marketed to target CD19 expressed on all B cells except plasma cells [31] and B cell maturation antigen (BCMA) expressed on memory B cells, plasmoblasts, and plasma cells [32]. CAR Tregs exert immunosuppressive effects to maintain immune homeostasis [12]. Currently, there are two directions for CAR immunotherapy for autoimmune diseases: one is to eliminate auto-reactive immune cells to deeply reset the immune system by CAR T cells, and the other is to inhibit auto-reactive immune cells by CAR Tregs [33]. Ongoing studies on the applications of CAR T cell therapy in CAR immunotherapy are presented in Table 3, with data from https://clinicaltrials.gov/.

**Table 3:** Ongoing clinical trials of CAR T cell therapy in non-oncological diseases

Disease	NCT no.	Antigen	Phase	Status	T cell source	Sponsor
SLE	NCT05030779	CD19/BCMA	Early Phase 1	Unknown	Unknown	Zhejiang University
	NCT06350110	CD19/BCMA	Phase 1/2	Recruiting	Autologous	Essen Biotech
	NCT06222853	CD19	Early Phase 1	Recruiting	Autologous	Zhejiang University
	NCT06340750	BAFF-R	Early Phase 1	Recruiting	Autologous	Luminary Therapeutics
	NCT06106906	CD19	Phase 1/2	Recruiting	Autologous	Wuhan Union Hospital
	NCT03030976	CD19	Phase 1	Unknown	Autologous	Shanghai GeneChem Company; RenJi Hospital
	NCT06310811	CD19	Not Applicable	Recruiting	Autologous	Wuhan Union Hospital
	NCT06333483	CD19	Phase 1	Recruiting	Autologous	Autolus Limited
	NCT06189157	CD19	Phase 1/2	Recruiting	Autologous	Miltenyi Biomedicine GmbH
	NCT05474885	CD19/BCMA	Phase 1	Recruiting	Autologous	iCell Gene Therapeutics; iCAR Bio Therapeutics Ltd China
	NCT05869955	CD19	Phase 1	Recruiting	Autologous	Juno Therapeutics, Inc., a Bristol Myers Squibb Company
	NCT05798117	CD19	Phase 1/2	Recruiting	Autologous	Novartis Pharmaceuticals
	NCT05858684	CD19/BCMA	Phase 1	Recruiting	Autologous	RenJi Hospital; Gracell Biotechnology Shanghai Company Ltd
	NCT06121297	CD19	Phase 1/2	Recruiting	Autologous	Cabaletta Bio
	NCT06297408	CD19	Phase 1	Not yet recruiting	Autologous	Shanghai Ming Ju Biotechnology Company Ltd
	NCT05846347	CD19/BCMA	Phase 1	Recruiting	Autologous	RenJi Hospital; Gracell Biotechnology Shanghai Company Ltd
	NCT05988216	CD19	Not Applicable	Recruiting	Allogeneic	Zhejiang University
SS	NCT05085431	CD19/BCMA	Early Phase 1	Recruiting	Autologous	Zhejiang University
SSc	NCT05085444	CD19/BCMA	Phase 1	Recruiting	Autologous	Zhejiang University
immunological nephritis	NCT05085418	CD19/BCMA	Early Phase 1	Recruiting	Autologous	Zhejiang University
	NCT06342960	CD19	Phase 1/2	Recruiting	Autologous	Kyverna Therapeutics
	NCT05938725	CD19	Phase 1	Recruiting	Autologous	Kyverna Therapeutics
MS	NCT06138132	CD19	Phase 1	Recruiting	Autologous	Stanford University; Kyverna Therapeutics
	NCT06220201	CD19	Phase 1	Recruiting	Autologous	Juno Therapeutics, Inc., a Bristol Myers Squibb Company; Celgene Corporation
	NCT06384976	CD19	Phase 2	Not yet recruiting	Autologous	Kyverna Therapeutics
MG	NCT06193889	CD19	Phase 2	Recruiting	Autologous	Kyverna Therapeutics
	NCT05828225	CD19	Phase 1	Recruiting	Autologous	Zhejiang University
	NCT05451212	MuSK	Phase 1	Recruiting	Autologous	Cabaletta Bio
	NCT04146051	BCMA	Phase 1/2	Recruiting	Autologous	Cartesian Therapeutics
IIM	NCT06154252	CD19	Phase 1/2	Recruiting	Autologous	Cabaletta Bio
	NCT06298019	CD19	Phase 1	Not yet recruiting	Autologous	Stanford University; Kyverna Therapeutics
AIHA	NCT06212154	CD19	Phase 1	Recruiting	Allogeneic	Institute of Hematology & Blood Diseases Hospital
autoimmune hemolytic anemia	NCT06231368	CD19	Phase 1	Recruiting	Autologous	Regenerative Medical Center, Institute of Hematology & Blood Diseases Hospital, China; Juventas Cell Therapy Ltd
	NCT06212154	CD19	Phase 1	Recruiting	Allogeneic	Institute of Hematology & Blood Diseases Hospital, China; Suzhou Fundamental Therapeutics
NMOSD	NCT05828212	CD19	Phase 1	Recruiting	Autologous	Zhejiang University
ITP	NCT06352281	Unknown	Phase 1/2	Recruiting	Autologous	920th Hospital of Joint Logistics Support Force of People’s Liberation Army of China
PV	NCT04422912	DSG3/CD19	Phase 1	Recruiting	Autologous	Cabaletta Bio
dermatomyositis	NCT06298019	CD19	Phase 1	Not yet recruiting	Autologous	Stanford University; Kyverna Therapeutics
Crohn’s disease	NCT05239702	CD7	Phase 1	Recruiting	Unknown	Zhejiang University
HIV	NCT03240328	HIV-1 envelope protein	Phase 1	Recruiting	Autologous	Sun Yat-sen University
	NCT06252402	gp120	Early Phase 1	Not yet recruiting	Autologous	City of Hope Medical Center
	NCT04648046	gp120	Phase 1/2	Recruiting	Autologous	University of California, San Francisco
	NCT03617198	gp120	Phase 1	active, not recruiting	Unknown	University of Pennsylvania

**Notes: SLE**, systemic lupus erythematosus; **SS**, Sjögren’s syndrome; **SSc**, systemic sclerosis; **MS**, multiple sclerosis; **MG**, myasthenia gravis; **AIHA**, autoimmune hemolytic anemia; **NMOSD**, neuromyelitis optica spectrum disorder; **ITP**, idiopathic thrombocytopenic purpura; **PV**, pemphigus vulgaris; **IIM**, Idiopathic inflammatory myopathies; **BAFF-R**, B cell activating factor receptor

#### Systemic lupus erythematosus (SLE)

SLE is a classic systemic autoimmune disease in which autoantibodies against double-stranded DNA and other nuclear antigens trigger inflammation in multiple organs through immune complex deposition [34–36]. B cell dysfunction and the production of autoantibodies are hallmark features of SLE [37, 38].

Early animal experiments have shown significant B cell depletion and improved disease symptoms in a mouse model of lupus following anti-CD19 CAR T cell treatment [39, 40]. Anti-CD19 CAR T has achieved promising results in the clinical treatment of SLE. Mougiakakos et al. [41] used anti-CD19 CAR T cells in a female patient with severe refractory SLE who did not respond to immunosuppressive therapy and found that the patient’s levels of double-stranded DNA autoantibodies and complement returned to normal, proteinuria improved, and the lupus disease activity index (SLEDAI) decreased from 16 to 0. Other experiments using anti-CD19 CAR T cells to treat SLE have also shown similar results [42, 43]. In addition, they found that re-emerging B cells had a naïve phenotype and non-class-switched B cell receptors, suggesting that immune reset effectively eliminated autoantibody-producing B cells [42]. The anti-CD19 CAR T showed good efficacy in treating SLE but did not target CD19-negative plasma cells. To address this limitation, many teams have utilized dual BCMA-CD19 compound CAR T cells or a combination of anti-CD19 CAR T and anti-BCMA CAR T therapies for SLE. In a clinical trial, 12 patients with SLE resistant to multiple immunosuppressive therapies received anti-CD19/BCMA CAR T cells, and all patients achieved a state of low disease activity in lupus and discontinued immunosuppressive medications [44]. CD19-BCMA compound CAR T cells significantly reduced the antibody levels, effectively targeting both B cells and plasma cells to ‘reset’ antibody-producing populations, resulting in a more comprehensive elimination of autoantibodies and enhancing clinical outcomes [45]. Wang et al. [46] *g*enerated BCMA-CD19 compound CAR T cells for 13 SLE patients and obtained similar results.

Anti-CD19 CAR Tregs are a promising therapeutic target. Doglio et al. [47] treated SLE mice with anti-CD19 CAR Tregs, which efficiently inhibited the proliferation and activity of B cells in vitro, limited autoantibody production, and delayed lymphopenia without detectable toxicity in vivo. Although preclinical studies have demonstrated the efficacy of CAR Tregs, prospective clinical trials are required to investigate their safety and efficacy.

#### Rheumatoid arthritis (RA)

RA is a chronic autoimmune disease characterized by synovial inflammation, with the main clinical manifestations being inflammatory lesions in the joints, which can occur at any age [48]. Treatment options are effective in alleviating symptoms and managing the progression of the disease [49].

Zhang et al. [50] developed a universal anti-fluorescein isothiocyanate (FITC) CAR T in combination with FITC-labeled antigenic peptides to eliminate the subset of self-reactive B cells that recognize these antigens in RA. The primary limitation of this approach is its demonstrated efficacy only in vitro for eliminating autoreactive B cells, with uncertainties regarding its toxicity to autoimmune B cells and its safety in vivo. Additionally, improvements are needed in the stability of autoantigens and FITC-coupled media [51]. A recent study [52] using anti-CD19 CAR T to treat one patient with both RA combined with myasthenia gravis (MG) suggested its potential effectiveness in achieving RA remission.

Citrullinated vimentin (CV) is exclusively present in the extracellular matrix of inflamed synovial tissue in patients with RA [53]. Raffin et al. [53] generated CV-specific CAR Tregs that were efficiently activated, expanded, and inhibited following CAR-mediated stimulation in vitro. The functional activity of CV-specific CAR Tregs in animal models is currently under investigation. Sonoma Biotherapeutics is developing CAR Treg therapies for RA [12].

#### Sjögren’s syndrome (SS)

SS is a chronic inflammatory autoimmune disease characterized by decreased secretion from the lacrimal and salivary glands [54]. The development of the disease is associated with multiple factors, including infection, endocrinology, and genetics, but the exact cause remains unclear [55–57].

Autoreactive B cells play a central role in SS. Selective eradication of autoreactive B cell clones is important for the treatment of SS [58]. A study [59] reported that a 76-year-old female patient with active SS for 10 years, combined with DLBCL, achieved complete remission (CR) by day 28 after anti-CD19 CAR T infusion. Antinuclear and anti-Ro-52 antibodies were negative on day 90, the patient maintained CR at six months with normalized serum cytokine levels, and improved dry mouth symptoms. The serological hallmark of primary SS is a high-titer IgG autoantibody against the autoantigen La/SSB, with LaA is a key epitope of the autoantigen La/SSB [60]. Meng et al. [58] produced LaA chimeric autoantibody receptor (CAAR) NK cells that can target the corresponding La/SSB-specific B cell receptor (BCR)-bearing lymphoma cells. These cases underscore the potential of CAR immunotherapy in treating autoimmune conditions like SS.

#### Demyelinating and neurodegenerative diseases

##### Multiple sclerosis (MS)

MS is an autoimmune condition characterized by inflammatory demyelinating lesions in the white matter of the central nervous system (CNS) [61]. MS is associated with autoreactive T cells that travel to the CNS and are activated by myelin antigens, such as myelin basic protein (MBP) and myelin oligodendrocyte glycoprotein (MOG), leading to myelin destruction and impaired neuromotor signaling [62]. High-dose glucocorticoid therapy is the recommended first-line treatment for MS [63].

B lymphocyte aggregates have been found in the meningeal compartments of patients with secondary progressive MS in response to ongoing inflammation [64]. CAR T targeting B cells may be beneficial for these patients. Anti-CD19 CAR T cells are currently used to treat experimental autoimmune encephalomyelitis (EAE) in animal models, with promising results [65, 66]. Fischbach et al. [67] reported therapeutic data using fully human CD19-targeted CAR T cell therapy in two patients with progressive multiple sclerosis, with reduced intrathecal antibody production in one of the patients following CAR T cell infusion. Further evaluation of the short- and long-term safety and therapeutic efficacy of this therapy is required.

A team performed experiments using anti-MBP/MOG CAR Tregs and found that these CAR Tregs could inhibit the proliferation of MBP-reactive T effector cells and ameliorate EAE induced by MOG [68]. Anti-MBP/MOG CAR Tregs from other teams have also shown similar effects in animal models [69, 70]. These experiments highlight the potential role of immunomodulatory engineered Tregs in reducing MS-related morbidity and mortality, an effort being pursued by several biopharmaceutical companies, such as Abata Therapeutics and TeraImmune [12].

##### Myasthenia gravis (MG)

MG is an autoimmune disorder characterized by acquired neuromuscular junction transmission dysfunction driven by autoantibodies directed against muscle-specific tyrosine kinase (MuSK), acetylcholine receptors, and other targets, potentially resulting in severe muscle weakness that can be life-threatening [71, 72].

A team designed MuSK CAAR T cells and found that MuSKCAAR T cells reduced anti-MuSK IgG levels but not total IgG levels, reflecting the depletion of MuSK-specific B cells in a mouse model [73]. Currently, CAR T therapy for patients with MG primarily targets B cells, using CD19/BCMA to “reset” antibody-producing populations. Haghikia et al. [74] used anti-CD19 CAR T cells to treat one patient with MG, and the patient’s symptoms improved. Granit et al. [75] included 14 patients with MG from eight regions in the U.S., who were treated with anti-BCMA CAR T cells, and all patients achieved clinical improvement. Tian et al. [76] administered autologous anti-BCMA CAR T cells to two patients with MG and none of the patients tested positive after their antibody levels were reduced to negative. Approximately 80% of the reconstituted B cells at 18 months in the two patients were naive B cells, whereas non-switched and switched memory B cells and plasma cells showed a decreasing trend. These findings suggest that reconstruction of the B cell lineage after CAR T cell treatment results in more primitive phenotypes and durable suppression of humoral immune responses.

##### Neuromyelitis Optica spectrum disorders (NMOSD)

NMOSD is characterized by inflammatory demyelination of the optic nerves and spinal cord, along with axonal damage, and is primarily associated with serum aquaporin-4 IgG antibodies [77].

Qin et al. pioneered a clinical study (NCT04561557) on the treatment of NMOSD with anti-BCMA CAR T cells and recruited 12 NMOSD patients [78]. During the median follow-up period, 11 patients were relapse-free and showed a decreasing trend in serum aquaporin-4 antibodies at the cutoff date. CAR T cells persisted in the peripheral blood for up to six months in 17% of the patients. IASO Biotherapeutics developed a fully human autologous anti-BCMA CAR T cell injection (Equecabtagene Autoleucel, equ-cel), which was formally approved by the Center for Drug Evaluation (CDE) of China’s National Medical Products Administration (NMPA) for an expanded indication in clinical trials for antibody-mediated NMOSD.

#### Idiopathic inflammatory myopathy (IIM)

IIM is a heterogeneous group of autoimmune diseases characterized by chronic myositis [79]. Based on the clinical and histopathological manifestations in muscle tissue, IIM is classified into the following subtypes: dermatomyositis, polymyositis, inclusion body myositis, antisynthetase syndrome (ASS), immune-mediated necrotizing myopathy (IMNM), cancer-associated myositis, and overlapping myositis [80].

ASS is characterized by typical autoantibodies against transfer RNA synthetases [81] and treating ASS with CARs that target B cells is a promising option. In one study, a patient with ASS treated with anti-CD19 CAR T cells showed rapid improvement in clinical symptoms and remission [81]. Other ASS patients treated with anti-CD19 CAR T have also shown similar results [82, 83]. Recently, allogeneic universal anti-CD19 CAR T therapy (TyU19) developed using CRISPR-Cas9 gene editing technology was successfully applied to treat one patient with IMNM and two patients with diffuse cutaneous systemic sclerosis (NCT05859997) [84]. TyU19 led to the depletion of B cells in these patients, attenuated the severity of the disease in the patients with refractory IMNM, and reversed extensive fibrotic damage to critical organs in two patients with diffuse cutaneous systemic sclerosis. Overall, these results indicate that CAR T cell therapy may reduce inflammation in the muscles, joints, and lungs of patients with IIM. However, continued monitoring of the long-term efficacy and safety of all patients is needed.

#### Dermatological diseases

##### Pemphigus vulgaris (PV)

Pemphigus is a rare autoimmune herpetic disease that affects the skin and mucous membranes and poses a serious threat to human health. PV is the most common and severe form of pemphigus [85]. The pathogenesis of PV involves anti-desmoglein (Dsg) antibodies that disrupt the epidermal cell junctions of a protein called desmoglein 3 (Dsg3), leading to clinical manifestations such as blisters and maculopapular rash [86]. In 2016, Ellebrecht et al. [87] generated Dsg3 CAAR T cells, which exhibited cytotoxicity against Dsg3-specific B cells in vitro and amplified, sustained, and specifically eliminated B cells expressing anti-Dsg3 BCR in vivo. Later, another team also designed Dsg3 CAAR T cells and showed similar results [88]. CAAR T cells may provide an effective and versatile strategy for the specific targeting of autoreactive B cells in antibody-mediated autoimmune diseases. However, further clinical trials are required to validate the efficacy of Dsg3 CAAR T cells in patients with PV.

##### Vitiligo

Vitiligo, with a prevalence of approximately 1%, is an autoimmune skin depigmentation disorder characterized by the appearance of white patches due to melanocyte destruction [89, 90]. Studies have shown that pigment loss is associated with T cell and macrophage infiltration of the dermis [91]. There is no systemic reduction in the abundance and activity of Tregs, but very few Tregs are found in the skin of patients with vitiligo [92]. Even when very few Tregs are transferred into vitiligo skin, they can interfere with depigmentation [93]. Both the adoptive transfer of Tregs and the use of rapamycin induce durable remission of vitiligo in mice [94]. Ganglioside D3 (GD3) is overexpressed in epidermal cells (including melanocytes) in vitiligo lesions [95]. A team produced GD3-specific CAR Tregs to treat vitiligo [96]. Mice from a model of spontaneous vitiligo that received GD3-specific Tregs showed increased secretion of IL-10, controlled cytotoxicity towards melanocytes, and a delay in pigmentation loss. CAR Tregs offer a new approach to the treatment of vitiligo.

#### Type 1 diabetes (T1D)

T1D is a metabolic disorder syndrome characterized by hyperglycemia due to insulin deficiency, which is caused by immune system-mediated destruction of pancreatic β cells [97]. It typically occurs during childhood and adolescence, has a rapid onset, and requires lifelong insulin therapy.

A team developed a monoclonal antibody (mAb287) targeting the I-Ag7-InsB9-23 antigenic complex as the extracellular structural domain of CAR T cells to selectively delete target antigen-presenting cells based on the binding of the insulin B-chain (InsB9-23) to an MHC class II molecule (I-Ag7) [98]. In this mouse model, a single infusion of CAR T cells to mice significantly delayed the onset of overt hyperglycemia. However, while CAR T cells delayed the development of T1D, they did not stop it. Tenspolde et al. [99] designed insulin-specific CAR Tregs that showed prolonged suppressive effects, functional stability, and longevity in vivo. In this study, insulin-specific CAR Tregs failed to prevent spontaneous diabetes in mice but persisted for up to four months. HPi2-CAR Tregs, which recognized the human pancreatic endocrine marker HPi2, failed to maintain expansion due to tonic signaling [100]. CAR Tregs against GAD65 Beta cell epitopes homed to the pancreatic islets of the humanized T1D mouse model at 24 h post-infusion. Compared with the control group, Treg populations in the pancreas and spleen were significantly increased, and blood glucose was lower in the CAR Tregs group [101]. Thus, CAR Tregs may help regain immune tolerance in patients with T1D.

#### Inflammatory bowel diseases (IBD)

IBD is triggered by various factors and is characterized by chronic inflammation of the gastrointestinal tract. Ulcerative colitis and Crohn’s disease are the most common forms of IBD [102].

A research team [103] designed a chimeric receptor that was transferred to Tregs expressing 2,4,6-trinitrophenol (TNP)-specific antibodies. They found that this receptor was resistant to 2,4,6-trinitrobenzenesulfonic acid-induced colitis, but not to other hapten-mediated colitis. These Tregs localized to the inflamed colonic mucosa in a mouse model of colitis, and bystander inhibited oxazolone-induced colitis. Due to the overexpression of carcinoembryonic antigen (CEA) in colitis, Blat et al. [104] designed CEA-specific CAR Tregs and applied them to a mouse model of colitis induced by azoxymethane-dextran sulfate sodium. Their results showed that CEA-specific CAR Tregs suppressed colitis while significantly reducing the burden of subsequent colorectal cancer tumors. Flagellin, a main structural protein of bacterial flagella, is an immunogenic antigen found in patients with colitis. One team [105] developed novel CAR Tregs targeting flagellin in *Escherichia coli* H18 (FliC). In a humanized mouse model, FliC-specific CAR Tregs migrated to damaged colon tissues, exhibited potent inhibitory effects, and promoted the establishment of a monolayer of colonic epithelial cells. These results broadly demonstrate the therapeutic potential of CARs targeting microbe-derived antigens.

#### Asthma

Asthma is a chronic respiratory condition characterized by airway hypersensitivity and fluctuating airflow constraints. IL-5-mediated eosinophil infiltration is an important pathological feature of type 2 asthma, and eosinophil-depleting antibodies have shown therapeutic efficacy in treating type 2 asthma [106].

One team constructed IL-5 CAR recognizing eosinophils that highly expressed the IL-5 receptor α [107]. This study found that IL-5 CAR T cells can achieve long-term efficacy in treating asthma with durable remission in mice. CEA is expressed on the cell surface of epithelial origin (e.g., adenoepithelia in the lungs and gastrointestinal tract) [108]. Skuljec et al. [109] designed CEA-specific CAR Tregs and found that these CAR Tregs accumulated in inflamed lungs, leading to decreased airway hyperreactivity, eosinophilic airway inflammation, mucus production, and lower levels of allergen-specific IgE and Th2 cytokines.

In autoimmune and inflammatory diseases, the goal is to eliminate disease-driving immune cell subsets through CAR T or reduce inflammation through redirected Tregs. Striking the right balance between these approaches is crucial for effective treatment. Treg-based strategies offer a broader, more tolerogenic solution but may lead to immune suppression. On the other hand, targeting disease-driving immune cells can provide a more focused treatment, though it may have broader implications for immune system function. Both approaches require careful control to minimize risks like increased infection or cancer susceptibility, while effectively managing the disease. CAR T cells can deplete B cells within the tissue and therefore demonstrate therapeutic efficacy in B cell-driven diseases such as SLE, MG, and MS. Tregs are suppressor cells that can inhibit T cells with different antigenic specificities through bystander suppression and induction of other suppressive cells by infectious tolerance [12]. CAR Tregs could offer a new therapeutic strategy for chronic inflammatory diseases that are not primarily driven by B cells, such as IBD, T1D, and asthma. CAR immunotherapy is primarily used to treat patients with severe diseases resistant to conventional therapies. In autoimmune diseases, most CAR T cells target CD19 and BCMA expressed on B cells or plasma cells [110]. In patients with autoimmune diseases, when CAR T cells disappear from the peripheral blood, B cells rebuild after a certain period [42, 111]. However, despite B cells reconstitution, autoantibodies and autoimmune disease manifestations do not recur. CAR T cell therapy can help to restore B cell homeostasis and even ‘reset’ the immune system, potentially leading to long-term remission [30, 42, 111, 112]. Although patients with autoimmune diseases treated with CAR immunotherapy have not experienced relapse yet, this risk still exists and requires long-term follow-up. The incidence of cytokine release syndrome (CRS) and immune cell-associated neurotoxicity syndrome (ICANS) is low in patients with autoimmune diseases, possibly due to the relatively low target cell loads [1].

### Infection

Infection is the process of harmful replication and reproduction of microorganisms, including viruses, bacteria, fungi, and other pathogens, within the host. The initial clinical trial of CAR T cells was conducted in individuals diagnosed with human immunodeficiency virus/acquired immunodeficiency syndrome (HIV/AIDS).

#### Virus

##### Human immunodeficiency virus (HIV)

The causative agent of AIDS is HIV, a highly mutable virus with the env gene having the highest rate of mutation [113]. HIV enters cells through receptors on the surface of susceptible cells, including the primary (CD4) and co-receptors (CCR5 or CXCR4) [113]. CAR T cell therapy was originally developed as a strategy for durable control of HIV [114].

CD4 is a ligand for the HIV gp120 protein. Mitsuyasu et al. [115] conducted a study using CD4 CAR T cells to treat 24 HIV-positive individuals. They observed a significant reduction in HIV RNA levels in rectal tissues, although no substantial change was seen in plasma HIV RNA levels. Deek et al. [116] conducted a phase II randomized trial with CD4 CAR T in 40 patients infected with HIV and observed no substantial differences in viral reservoirs between the study groups. Scholler et al. [117] conducted a clinical trial (NCT01013415) to assess CD4 CAR T cells in HIV and found that CD4 CAR T cells were detected in 98% of the samples tested for at least 11 years after infusion. The gp120 protein in HIV can bind to CD4, indicating that CD4 CAR T cells are vulnerable to HIV infection. To address this constraint, several studies have been conducted to prevent infection by modifying CD4 CAR T cells with CCR5-targeting zinc finger nucleases [118, 119], deleting the CCR5 gene through gene editing techniques [120], or designing broadly neutralizing antibodies (bNAbs) that target different epitopes on the HIV envelope protein, including the CD4 binding site, outer proximal region of the glycoprotein 41 (gp41) membrane, and variable region glycans [121–124]. Mao et al. [125] treated 18 HIV-1 patients with anti-HIV-1 CAR T cells armed with bNAbs and the follicle-homing receptor CXCR5, finding an average decrease in viral load of 67.1%, with 10 patients showing a sustained reduction in cell-associated HIV-1 RNA levels during the 150-day observation period. Another clinical trial (NCT03240328) that recruited 14 patients infected with HIV for a single infusion of bNAb-derived CAR T cells demonstrated similar results [126].

HIV arose from the zoonotic transfer of simian immunodeficiency virus (SIV) from primates to humans [127]. Programmed cell death protein 1 (PD-1) is an immune checkpoint marker normally expressed on memory T cells and is enriched in latent HIV-infected CD4 T cells [128, 129]. Eichholz et al. [129] generated anti-PD-1 CAR T cells for SIV-infected rhesus monkeys and observed the depletion of PD-1 + T cells in the blood and tissues as well as the eradication of SIV.

Bispecific CARs have been developed that fuse CD4 fragments to bNAb-based scFv or the carbohydrate-recognition structural domain of human C-type lectin [130–132]. Tri-specific CARs targeting three sites simultaneously have also been developed, such as CD4-MBL-R5Nt CARs [133]. The ongoing CAR immunotherapy studies for HIV infection are presented in Table 3.

##### Hepatitis B virus (HBV)

HBV is the causative agent of hepatitis B and is primarily transmitted through the blood, from mother to child and through sexual contact [123]. There are three HBV antigens: the e antigen (HBeAg), core antigen (HBcAg), and surface antigen (HBsAg) [134]. Current research focuses on CAR T cells engineered to target HBsAg. Bohne et al. [135] designed CAR T cells against HBsAg, which selectively eliminated HBV-infected target cells and cccDNA-positive target cells. Krebs et al. [136] and Kruse et al. [137] also developed similar CAR T for HBV and both showed the similar results as described above. However, hepatotoxicity may pose a safety concern, and protective mechanisms for the on-demand depletion of transferred T cells are required. Klopp et al. [138] designed caspase 9 or herpes simplex virus thymidine kinase as safety switches to reduce CRS response and hepatotoxicity. They found that the activation of both safety switches stopped cytotoxicity after less than 1 h. CAR immunotherapy offers another new treatment for HBV-resistant patients, but side effects such as hepatotoxicity should still be noted.

##### Hepatitis C virus (HCV)

HCV is an RNA virus that selectively targets hepatocytes. The HCV genome contains three structural proteins (core, E1, and E2) and seven nonstructural proteins (P7, NS2, 3, 4 A, 4 B, 5 A, and 5B) that are crucial for HCV replication and spread and are therefore likely to be potential targets for antiviral drugs [139]. In 2014, Sautto et al. [140] designed anti-HCV/E2 CAR T cells, which recognized specific antigens, lysed HCV-infected hepatocytes, and secreted interferon γ, interleukin 2, and tumor necrosis factor α. However, the HCV/E2 protein is highly susceptible to mutations, and the exploration of other conserved antigens is necessary.

##### Human cytomegalovirus (HCMV)

HCMV is a DNA virus classified within the Herpesviridae family that encodes five proteins with anti-apoptotic activity (UL36, UL37 × 1, UL37 medium protein, UL37 glycoprotein, and UL38) and an untranslated beta 2.7 RNA [141]. HCMV-encoded glycoprotein B (gB) is highly expressed in cell membranes shortly following HCMV infection, reaching a peak at 72–96 h post-infection [142]. Olbrich et al. [143] designed anti-gB CAR T cells, which demonstrated strong targeting effects in vitro. Proff et al. [144] also developed anti-gB CAR T cells that could recognize HCMV-infected cells and release cytokines and cytotoxic particles. However, cells infected with HCMV showed resistance to anti-gB CAR T cells, which may be related to the functions of the viral anti-suicide machinery, such as the expression of proteins UL37 × 1 and UL36.

##### Epstein-Barr virus (EBV)

EBV is a latent oncogenic herpesvirus linked to various malignancies. The envelope proteins of EBV, such as gB and gp350, are crucial for facilitating EBV entry and infection of target cells [145]. EBV can express a restricted array of viral proteins, including latent membrane proteins (LMP1 and LMP2) [146]. These proteins are potential targets for EBV treatment. Tang et al. [147] constructed LMP1-specific CAR T cells that secreted IFN-γ and IL-2, specifically killing LMP-expressing cells. In 2020, Slabik et al. [148] designed anti-gp350 CAR T cells that targeted and eliminated EBV-infected cells *in vitro.* These CAR T cells also reduced EBV dissemination, decreased the frequency of EBER B cell malignant lymphocyte proliferation, and alleviated inflammation in 75% of treated mice. However, 25% of mice treated with anti-gp350 CAR T cells showed increased EBV infection, which may be related to the immune escape mechanism of EBV. The immune escape mechanisms warrant further attention. Other potential target EBV glycoproteins, such as gB, gH, gL and gp42, could be the new targets and combinations to prevent immune escape mechanisms.

##### Coronavirus disease 2019 (COVID-19)

COVID-19 can cause moderate to severe life-threatening illnesses and organ dysfunction [149]. The viral envelope surface contains spike protein [150]. Ma et al. [151]. developed anti-spike CAR NK cells that specifically bound to cells infected with SARS-CoV-2 and eliminated them in vitro. Christodoulou et al. [152] produced the H84T-Banana Lectin CAR NK cells, which bind high-mannose glycans near the receptor-binding region of spike proteins on the viral envelope and exerts antiviral effects. NKG2D serves as a broadly activating receptor on NK cells that recognizes infected cells, whereas ACE2 is a known receptor that binds to SARS-CoV-2 spike proteins. A clinical trial (NCT04324996) investigated bispecific NKG2D/ACE2 CAR NK cells as potential therapeutic targets for COVID-19.

#### Fungi

##### Aspergillus Fumigatus

*Aspergillus fumigatus* (Af), whose cell wall consists mainly of glucan, chitin, and galactomannan, is the primary causative agent of invasive pulmonary aspergillosis, typically seen in immunocompromised populations [153]. Dectin-1 is a C-type lectin receptor expressed in macrophages, neutrophils, and dendritic cells, and it binds specifically to β-glucan in fungal cell walls [154]. A team developed dectin-1 CAR T cells, which impaired and inhibited the growth of Aspergillus filaments both in vitro and in vivo [155]. Seif et al. [153] produced an anti-Af CAR T that specifically recognized *Aspergillus fumigatus* and released perforin and granzyme B to kill the fungus without affecting the human tissues. However, the fungal cell wall antigens recognized by anti-Af CAR remain unknown and need further exploration.

##### Cryptococcus neoformans

*Cryptococcus neoformans* is a conditionally pathogenic fungus that is ubiquitous in the environment and can cause severe infections of the central nervous system. The outer layer of *Cryptococcus neoformans* is unique in that it consists of three components: the cell membrane, cell wall, and capsule, which is primarily composed of glucuronoxylomannan (GXM)—considered the main virulence factor [156]. Silva et al. [157] proposed anti-GXM CAR T cells that could specifically target *Cryptococcus neoformans*, control its growth, and reduce fungal load. This study suggests a potential new therapy for cryptococcosis.

CAR immunotherapy targets non-human antigens in infections and can eliminate microbiologically infected cells from the body, offering a new therapeutic avenue for the treatment of infections. In infectious diseases, CARs specifically recognize antigens of pathogenic microorganisms and thus exert the killing effects. However, for microbial infections of vital organs, CAR T cell therapy may lead to organ damage. For example, anti-HBsAg CAR T cells eliminate HBV-infected liver cells, which can cause liver injury. The cost of CAR immunotherapy is high, but common anti-infective drugs are relatively inexpensive and can be very effective for common infectious diseases. Current research on CAR immunotherapy for infections has focused on viruses and fungi, with relatively few studies on bacteria—likely due to the high efficacy of antibiotics. However, CARs have also been proposed for the treatment of *Mycobacterium tuberculosis* [158]. CAR immunotherapy is now being explored as a potential treatment for drug-resistant and refractory infectious diseases.

### Fibrosis

Fibrosis is not a disease but rather a highly dynamic process that results from dysregulated tissue repair responses to various types of tissue injury, particularly during chronic inflammatory disease processes [159]. CAR immunotherapy is currently being explored for the treatment of fibrosis, especially in the field of cardiac and hepatic fibrosis.

#### Cardiac fibrosis

Cardiomyocytes are non-renewable cells [160]. Therefore, when the heart suffers loss, it can only be repaired by fibrosis, and the resulting fibrotic scarring can interfere with cardiac function [161]. While there are no specific anti-fibrotic drugs, CAR immunotherapy presents a novel approach in the treatment of myocardial fibrosis.

Fibroblast activation protein (FAP), an endogenous protein expressed by activated fibroblasts, is rarely found in normal human hearts but highly expressed in pathological diseases such as DCM/HCM [162]. A team designed FAP-targeted CAR T cells for use in mice model and found a significant reduction in cardiac fibrosis, partial recovery of cardiac systolic and diastolic function, and few immunotoxic side effects [162]. The team designed a CD5/LNP-FAP CAR T cell, which, due to its inability to genomically integrate, persists in vivo for only a short duration [163]. Significant improvement in the function of fibrotic mice treated with in vivo-produced transient FAP CAR T cells was also observed. These findings open new opportunities for CAR immunotherapy as an anti-fibrotic treatment.

#### Hepatic fibrosis

Liver fibrosis is the diffuse over-deposition and abnormal distribution of the extracellular matrix in the liver, which is the pathological repair response of the liver to chronic injury [164]. Treatments directly targeting fibrosis remain limited.

The urokinase-type plasminogen activator receptor (uPAR) stimulates intracellular signaling and regulates both physiological processes and pathological events such as inflammation and tumor progression [165, 166]. High uPAR expression has been observed in liver fibrosis and uPAR-specific CAR T cells have ameliorated hepatic fibrosis induced chemically or by diet in mice [166, 167]. Given macrophages’ unique intrinsic properties and ability to engraft in the liver, Dai et al. [168] constructed anti-uPAR CAR macrophages and found that liver fibrosis was reduced and liver function was restored in a mouse model of liver fibrosis. Additionally, FAP was shown to be upregulated in pathologically fibrotic tissues, which may also be a suitable target for hepatic fibrosis [162, 169–171].

Fibrosis is a significant clinical burden that requires new therapeutic approaches. In fibrotic diseases, CARs can specifically recognize pathologically elevated antigens and exert their effects. CAR immunotherapy has demonstrated anti-fibrotic efficacy in murine models of hepatic and cardiac fibrosis. Although CRS has not been reported in mouse models, clinical data on CAR T cell therapy for the cancer treatment suggest that cytotoxicity associated with CRS is one of the major side effects of this therapy in humans. This issue may be alleviated by using CAR NK cells [172]. CAR immunotherapy is expected to be suitable for other fibrotic diseases.

### Hemophilia

Hemophilia is classified as hemophilia A or B based on factor VIII (FVIII) or factor IX (FIX) deficiency [173]. The disease requires the frequent use of clotting factors, which may trigger a drug-resistant immune response. Researchers have developed strategies using Tregs and chimeric antigen receptor technology to promote tolerance to coagulation factors [12]. Parvathaneni et al. [174] engineered FVIII CAR T cells that effectively killed FVIII-specific B-cell hybridomas and prevented the formation of anti-FVIII antibodies in hemophilic mice in vivo. Yoon et al. [175]. engineered an anti-FVIII CAR Tregs which proliferated in response to FVIII, inhibited the proliferation of FVIII-specific T effector cells, and prevented FVIII-specific antibodies in vitro, suggesting bystander suppression. The ectopic expression of FoxP3 produces potent and functional Tregs that inhibit antibody production against FVIII in vivo [176]. Herzog et al. [177]. administered FVIII-specific CD4 + FoxP3 + cells to hemophilia A mice and found that the production of inhibitory antibodies in response to FVIII protein administration was completely inhibited.

### Senescence

Cellular senescence entails the cessation of the cell cycle and secretion of factors that modulate the tissue microenvironment. Physiologically, this mechanism acts as a natural defense against tumors by utilizing a senescence-associated secretory phenotype to recruit T and NK cells from the immune system, thereby facilitating the removal of senescent cells [178]. However, pathologically, the accumulation of senescent cells can lead to an inflammatory state that contributes to ongoing tissue damage and a range of senescence-related diseases, such as liver and lung fibrosis, atherosclerosis, diabetes, and osteoarthritis [167, 179].

uPAR is a cell surface protein whose expression increases widely during senescence [180]. Amor et al. [167]. developed a senolytic therapy with anti-uPAR CAR T cells and found that it was effective in clearing senescent cells from animals and reversing liver fibrosis. This study confirms the potential of CAR T cells in addressing age-related conditions. The team reported that anti-uPAR CAR T cells ameliorated glucose tolerance and exercise capacity in both physiological aging and metabolic syndrome models, alleviating metabolic dysfunction [181]. NKG2D ligands (NKG2DLs) are highly expressed in senescent but not normal cells [182]. Yang et al. [183]. designed NKG2D CAR T cells that selectively eliminated senescent cells expressing NKG2DLs in vivo in aged mice and non-human primates, reversing aging-related phenotypes. MICA, MICB, ULBP1-5, and GPNMB expressed in senescent cells, may also serve as potential targets for CAR T cells [183]. The aforementioned experiments suggest that CAR T cell therapy holds promise for the treatment of age-related diseases.

### Transplant rejection and GvHD

Organ transplantation is the most promising treatment for patients with end-stage organ failure [184]. Immunosuppressants play a crucial role in organ transplantation [185, 186]. The use of CARs in transplantation has markedly shifted from chemical immunosuppressive strategies to cell-based therapies.

Mo et al. [187]. engineered CAR T cells against OX40, a co-stimulatory receptor that is elevated in circulating T cells during GvHD, and selectively eliminated OX40 + T cells. This study introduces a novel therapeutic strategy for GvHD. Tregs have emerged as important components of GvHD [12]. Using anti-MHC I CAR Tregs, they extended the survival of islet allografts and induced alloantigen-specific tolerance in secondary skin grafts [188]. HLA-A2 is a commonly mismatched antigen. MacDonald et al. [189] generated HLA-A2-specific CAR Tregs that prevented heterologous GvHD in immunocompromised mice. HLA-A2-specific CAR Tregs completely blocked immune reactions of allogeneic HLA-A2 + target cells and tissues [190]. HLA-A2-specific CAR Tregs attenuate alloimmune-mediated skin damage in mice, showing effects similar to those previously reported [191]. CAR Tregs are currently an important area of treatment for GvHD and prevention of allograft rejection, and the CAR Tregs trial (NCT04817774) for kidney transplantation has been authorized [12, 19]. Donor tissues and organs contain different HLA molecules that can be ideal therapeutic targets [19, 192].

## Challenges

CAR immunotherapy for the treatment of oncological and non-oncological diseases has similarities. Both approaches involve genetic engineering to modify cells so they can specifically identify and kill target cells, and both are personalized therapies. In oncology treatments, CARs target tumor cells, whereas in non-oncology treatments, CARs target immune cells, inflammatory cells, or pathogenic microorganisms.

CAR immunotherapy still faces safety challenges in non-oncology areas, such as short-term safety (e.g. ICANS, CRS), long-term safety (e.g. relapse, secondary malignancies, hematologic toxicity). However, the side effects of CARs are less severe in non-oncological diseases compared to tumor treatments. In non-oncological areas, such as autoimmune diseases, mild CRS and rare ICANS have been observed in clinical trials, which may be related to the low patient inclusion rates in these trials and the relatively low load of target cells in non-oncological diseases. Due to the potential for significant adverse effects, patients undergoing CAR T cell therapy must have sufficient organ reserve to tolerate these acute complications. CAR T cell therapy may not be the optimal choice for mild to moderate autoimmune diseases that can be effectively managed with conventional medications. Since CAR T cells primarily target B cells, they do not address the pathological T cells involved in autoimmune diseases. Furthermore, anti-CD19 CAR T cells do not eliminate CD19-negative plasma cells, which can produce autoantibodies, potentially leading to relapse of the autoimmune condition. An increasing concern with CAR T cell therapy is the potential risk of secondary cancers, especially T cell neoplasms linked to viral vector integration. Nearly all cases consistently report a secondary tumor incidence of less than 5% following CAR T therapy [193], and these secondary tumors may be associated with factors such as random insertion of viral vectors and epigenetic abnormalities [194]. Although an association between CAR T therapy and the development of secondary tumors has been observed, causality remains largely unproven, with the exception of rare cases of transgene-positive T cell lymphoma [195]. In January 2024, the FDA issued safety labeling change letters directly to six marketed CAR T products, requiring them to add a black box warning about the risk of T cell malignancy. Although there is no evidence that patients with autoimmune diseases treated with CAR T cells are at high risk of developing secondary malignancies, active monitoring for secondary malignancies after CAR T infusion is necessary. Given the risks of relapse, secondary malignancies and hematologic toxicities, such as neutropenia, long-term monitoring of these patients is essential. To address the cytotoxicity, CAR structures can be modified, CAR-transduced T cells can be altered, and ‘off-switches’ or suicide gene strategies can be added [196].

CAR immunotherapy in the non-oncology field still has challenges in targeting normal physiological cells. CARs for autoimmune diseases primarily target CD19/BCMA expression in B cells to treat the disease by therapeutically removing normal cell subpopulations, and their long-term efficacy requires continued follow-up. CAR treatment for aging and fibrosis targets proteins whose expression is increased in pathological cells but is also expressed in other physiological cells, and the exact negative impacts will need to be monitored. However, for infectious diseases, such as fungi, CARs target fungal cell walls that are not present in normal human body, suggesting that CARs do not show off-target effects on physiological cells. Unlike tumors, cells involved in non-oncological diseases cannot simply be removed by CAR T cells, as these cells may have indispensable physiological functions. Therefore, it is important to select appropriate targets for non-oncological diseases. The expression of antigens in non-oncological diseases may not be constant. For example, the HCV/E2 protein is highly susceptible to mutations, and the exploration of other conserved antigens is necessary. Therefore, it is important to identify novel target antigens for non-oncological diseases.

CAR immunotherapy still faces genotoxic risks. CAR T cells are typically generated through viral gene transfer methods, which enable stable integration of the CAR transgene. This ensures continuous expression of the CAR gene, thereby enhancing the T cells’ ability to recognize and attack cancer cells. However, the integration process can lead to undesirable genetic mutations or genomic instability, potentially resulting in long-term genotoxic risks. In contrast, non-viral gene transfer methods produce only transient expression of the transgene. These methods tend to have lower genotoxicity and may reduce the risk of mutations or autoimmunity. As such, they could offer particular advantages in non-cancer applications, such as autoimmune diseases.

The clinical translation of CAR immunotherapy in non-oncology areas is limited by cost, accessibility and production scalability, with the exception of autoimmune diseases. CAR immunotherapy, with its complex and time-consuming production process, is expensive. Accessibility to CAR immunotherapy is limited due to regional disparities and differences in healthcare infrastructure. The scalability of CAR T production is also constrained by its individualized nature, as it often requires the extraction and customization of T cells from the patients, along with the complex and time-consuming production process and limitations in production capacity. These factors collectively hinder the broader clinical translation of CAR immunotherapy, but they may be solved through automated processes, off-the-shelf products, and decentralized production. CAR immunotherapy is costly, but common anti-infective drugs are relatively inexpensive and very effective against common infectious diseases. However, for hard-to-treat infections, such as fungal infections and tuberculosis, CAR immunotherapy offers a good option.

## Conclusion

Currently, there is substantial experimental and clinical research on CAR immunotherapy targeting non-oncological diseases with promising results. However, its limitations in safety, clinical translation, and target selection indicate that further research, optimization, and clinical validation are needed to unlock its full potential. The limitations of current data include limited clinical evidence, insufficient long-term follow-up, and complexity in target antigens. There is a lack of large-scale trials and long-term follow-up to evaluate efficacy and safety in non-oncological diseases. Future research should aim to expand clinical trials, identify new targets, improve safety, and enhance the cost-effectiveness of CAR T for non-oncological diseases.

## Data Availability

No datasets were generated or analysed during the current study.

## Abbreviations

CAR: Chimeric antigen receptor
CAAR: Chimeric autoantibody receptor
scFv: Single-chain variable fragment
GvHD: Graft-versus-host disease
ECM: Extracellular matrix
OVA: Ovalbumin peptide
FAP: Fibroblast activation protein
DCM/HCM: Dilated cardiomyopathy/hypertrophic cardiomyopathy
uPAR: Urokinase-type plasminogen activator receptor
NASH: Non-alcoholic steatohepatitis
SLE: Systemic lupus erythematosus
RA: Rheumatoid arthritis
SS: Sjögren’s syndrome
SSc: Systemic sclerosis
MS: Multiple sclerosis
MG: Myasthenia gravis
AIHA: Autoimmune hemolytic anemia
NMOSD: Neuromyelitis optica spectrum disorders
ITP: Idiopathic thrombocytopenic purpura
mPV: Mucosal-dominant pemphigus vulgaris
BAFF-R: B cell activating factor receptor
DLBCL: Diffuse large B-cell lymphoma
RF: Rheumatoid factor
CCP: Anti-cyclic citrullinated polypeptide
FITC: Fluorescein isothiocyanate
CII: Type II collagen
IIM: Idiopathic inflammatory myopathies
DM: Dermatomyositis
PM: Polymyositis
IBM: Inclusion body myositis
ADM: Anaplastic dermatomyositis
ASS: Antisynthetase syndrome
IMNM: Immune-mediated necrotising myopathy
ICANS: Neurotoxicity syndrome
MBP: Myelin basic protein
EAE: Encephalomyelitis
MOG: Myelin oligodendrocyte glycoprotein
Dsg: Desmoglein
MuSK MG: Muscle-specific tyrosine kinase myasthenia gravis
AIDS: Acquired immunodeficiency syndrome
GXM: Glucuronoxylomannan
NKG2DLs: Natural killer group 2 member D ligands
HIV/AIDS: Human immunodeficiency virus/acquired immunodeficiency syndrome
bNAbs: Broadly neutralizing antibodies
HBV: Hepatitis B virus
HCV: Hepatitis C virus
HCMV: Human cytomegalovirus
EBV: Epstein-Barr virus
HSCT: Hematopoietic stem cell transplantation

## References

[1] Baker DJ, Arany Z, Baur JA, Epstein JA, June CH. CAR T therapy beyond cancer: the evolution of a living drug. Nature. 2023;619(7971):707–15. 37495877 10.1038/s41586-023-06243-w PMC12522170

[2] Utkarsh K, Srivastava N, Kumar S, Khan A, Dagar G, Kumar M, et al. CAR-T cell therapy: a game-changer in cancer treatment and beyond. Clin Translational Oncology: Official Publication Federation Span Oncol Soc Natl Cancer Inst Mexico. 2024;26(6):1300–18. 10.1007/s12094-023-03368-2 38244129

[3] Hughes-Parry HE, Cross RS, Jenkins MR. The evolving protein Engineering in the design of Chimeric Antigen Receptor T Cells. Int J Mol Sci. 2019;21(1). 10.3390/ijms21010204 PMC6981602 31892219

[4] Mazinani M, Rahbarizadeh F. CAR-T cell potency: from structural elements to vector backbone components. Biomark Res. 2022;10(1):70. 36123710 10.1186/s40364-022-00417-w PMC9487061

[5] Rafiq S, Hackett CS, Brentjens RJ. Engineering strategies to overcome the current roadblocks in CAR T cell therapy. Nat Reviews Clin Oncol. 2020;17(3):147–67. 10.1038/s41571-019-0297-y PMC7223338 31848460

[6] Eshhar Z, Waks T, Gross G, Schindler DG. Specific activation and targeting of cytotoxic lymphocytes through chimeric single chains consisting of antibody-binding domains and the gamma or zeta subunits of the immunoglobulin and T-cell receptors. Proc Natl Acad Sci USA. 1993;90(2):720–4. 8421711 10.1073/pnas.90.2.720 PMC45737

[7] Kowolik CM, Topp MS, Gonzalez S, Pfeiffer T, Olivares S, Gonzalez N, et al. CD28 costimulation provided through a CD19-specific chimeric antigen receptor enhances in vivo persistence and antitumor efficacy of adoptively transferred T cells. Cancer Res. 2006;66(22):10995–1004. 17108138 10.1158/0008-5472.CAN-06-0160

[8] Caruana I, Weber G, Ballard BC, Wood MS, Savoldo B, Dotti G. K562-Derived whole-cell vaccine enhances antitumor responses of CAR-Redirected virus-specific cytotoxic T lymphocytes in vivo. Clin cancer Research: Official J Am Association Cancer Res. 2015;21(13):2952–62. 10.1158/1078-0432.CCR-14-2998 PMC4490027 25691731

[9] Pan K, Farrukh H, Chittepu V, Xu H, Pan CX, Zhu Z. CAR race to cancer immunotherapy: from CAR T, CAR NK to CAR macrophage therapy. J Experimental Clin cancer Research: CR. 2022;41(1):119. 10.1186/s13046-022-02327-z PMC8969382 35361234

[10] Liu E, Marin D, Banerjee P, Macapinlac HA, Thompson P, Basar R, et al. Use of CAR-Transduced Natural Killer cells in CD19-Positive lymphoid tumors. N Engl J Med. 2020;382(6):545–53. 32023374 10.1056/NEJMoa1910607 PMC7101242

[11] Klichinsky M, Ruella M, Shestova O, Lu XM, Best A, Zeeman M, et al. Human chimeric antigen receptor macrophages for cancer immunotherapy. Nat Biotechnol. 2020;38(8):947–53. 32361713 10.1038/s41587-020-0462-y PMC7883632

[12] Arjomandnejad M, Kopec AL, Keeler AM. CAR-T Regulatory (CAR-Treg) cells: Engineering and Applications. Biomedicines. 2022;10(2). 10.3390/biomedicines10020287 PMC8869296 35203496

[13] Rozenbaum M, Meir A, Aharony Y, Itzhaki O, Schachter J, Bank I, et al. Gamma-Delta CAR-T cells Show CAR-Directed and Independent Activity against Leukemia. Front Immunol. 2020;11:1347. 32714329 10.3389/fimmu.2020.01347 PMC7343910

[14] Gong Y, Klein Wolterink RGJ, Wang J, Bos GMJ, Germeraad WTV. Chimeric antigen receptor natural killer (CAR-NK) cell design and engineering for cancer therapy. J Hematol Oncol. 2021;14(1):73. 33933160 10.1186/s13045-021-01083-5 PMC8088725

[15] Xie G, Dong H, Liang Y, Ham JD, Rizwan R, Chen J. CAR-NK cells: a promising cellular immunotherapy for cancer. EBioMedicine. 2020;59:102975. 32853984 10.1016/j.ebiom.2020.102975 PMC7452675

[16] Yoon DH, Osborn MJ, Tolar J, Kim CJ. Incorporation of Immune Checkpoint Blockade into Chimeric Antigen Receptor T Cells (CAR-Ts): combination or Built-In CAR-T. Int J Mol Sci. 2018;19(2). 10.3390/ijms19020340 PMC5855562 29364163

[17] Chen Y, Yu Z, Tan X, Jiang H, Xu Z, Fang Y, et al. CAR-macrophage: a new immunotherapy candidate against solid tumors. Volume 139. Biomedicine & pharmacotherapy = Biomedecine & pharmacotherapie; 2021. p. 111605. 10.1016/j.biopha.2021.111605 33901872

[18] Liu W, Putnam AL, Xu-Yu Z, Szot GL, Lee MR, Zhu S, et al. CD127 expression inversely correlates with FoxP3 and suppressive function of human CD4 + T reg cells. J Exp Med. 2006;203(7):1701–11. 16818678 10.1084/jem.20060772 PMC2118339

[19] Eskandari SK, Daccache A, Azzi JR. Chimeric antigen receptor T(reg) therapy in transplantation. Trends Immunol. 2024;45(1):48–61. 38123369 10.1016/j.it.2023.11.005

[20] Abreu TR, Fonseca NA, Gonçalves N, Moreira JN. Current challenges and emerging opportunities of CAR-T cell therapies. J Controlled Release: Official J Controlled Release Soc. 2020;319:246–61. 10.1016/j.jconrel.2019.12.047 31899268

[21] Abate-Daga D, Lagisetty KH, Tran E, Zheng Z, Gattinoni L, Yu Z, et al. A novel chimeric antigen receptor against prostate stem cell antigen mediates tumor destruction in a humanized mouse model of pancreatic cancer. Hum Gene Ther. 2014;25(12):1003–12. 24694017 10.1089/hum.2013.209 PMC4270113

[22] Majzner RG, Mackall CL. Tumor Antigen escape from CAR T-cell therapy. Cancer Discov. 2018;8(10):1219–26. 30135176 10.1158/2159-8290.CD-18-0442

[23] Sterner RC, Sterner RM. CAR-T cell therapy: current limitations and potential strategies. Blood cancer J. 2021;11(4):69. 33824268 10.1038/s41408-021-00459-7 PMC8024391

[24] Ramello MC, Benzaïd I, Kuenzi BM, Lienlaf-Moreno M, Kandell WM, Santiago DN et al. An immunoproteomic approach to characterize the CAR interactome and signalosome. Sci Signal. 2019;12(568). 10.1126/scisignal.aap9777 PMC6506216 30755478

[25] Koneru M, Purdon TJ, Spriggs D, Koneru S, Brentjens RJ. IL-12 secreting tumor-targeted chimeric antigen receptor T cells eradicate ovarian tumors in vivo. Oncoimmunology. 2015;4(3):e994446. 25949921 10.4161/2162402X.2014.994446 PMC4404840

[26] Depil S, Duchateau P, Grupp SA, Mufti G, Poirot L. Off-the-shelf’ allogeneic CAR T cells: development and challenges. Nat Rev Drug Discovery. 2020;19(3):185–99. 31900462 10.1038/s41573-019-0051-2

[27] Valdes AZ. Chapter 17 - immunological tolerance and autoimmunity. In: Rezaei N, editor. Translational autoimmunity. Volume 1. Academic; 2022. pp. 325–45.

[28] Wang L, Wang FS, Gershwin ME. Human autoimmune diseases: a comprehensive update. J Intern Med. 2015;278(4):369–95. 26212387 10.1111/joim.12395

[29] Fugger L, Jensen LT, Rossjohn J. Challenges, Progress, and prospects of developing therapies to treat Autoimmune diseases. Cell. 2020;181(1):63–80. 32243797 10.1016/j.cell.2020.03.007

[30] Schett G, Mackensen A, Mougiakakos D. CAR T-cell therapy in autoimmune diseases. Lancet (London England). 2023;402(10416):2034–44. 37748491 10.1016/S0140-6736(23)01126-1

[31] Wang K, Wei G, Liu D. CD19: a biomarker for B cell development, lymphoma diagnosis and therapy. Experimental Hematol Oncol. 2012;1(1):36. 10.1186/2162-3619-1-36 PMC3520838 23210908

[32] Martin J, Cheng Q, Laurent SA, Thaler FS, Beenken AE, Meinl E et al. B-Cell Maturation Antigen (BCMA) as a biomarker and potential treatment target in systemic Lupus Erythematosus. Int J Mol Sci. 2024;25(19). 10.3390/ijms251910845 PMC11476889 39409173

[33] Blache U, Tretbar S, Koehl U, Mougiakakos D, Fricke S. CAR T cells for treating autoimmune diseases. RMD open. 2023;9(4). 10.1136/rmdopen-2022-002907 PMC10668249 37996128

[34] Durcan L, O’Dwyer T, Petri M. Management strategies and future directions for systemic lupus erythematosus in adults. Lancet (London England). 2019;393(10188):2332–43. 31180030 10.1016/S0140-6736(19)30237-5

[35] Fava A, Petri M. Systemic lupus erythematosus: diagnosis and clinical management. J Autoimmun. 2019;96:1–13. 30448290 10.1016/j.jaut.2018.11.001 PMC6310637

[36] Lisnevskaia L, Murphy G, Isenberg D. Systemic lupus erythematosus. Lancet (London England). 2014;384(9957):1878–88. 24881804 10.1016/S0140-6736(14)60128-8

[37] Gómez-Bañuelos E, Fava A, Andrade F. An update on autoantibodies in systemic lupus erythematosus. Curr Opin Rheumatol. 2023;35(2):61–7. 36695053 10.1097/BOR.0000000000000922 PMC9881844

[38] Stojkic I, Harper L, Coss S, Kallash M, Driest K, Lamb M et al. CAR T cell therapy for refractory pediatric systemic lupus erythematosus: a new era of hope? Pediatric rheumatology online journal. 2024;22(1):72. 10.1186/s12969-024-00990-4 PMC11308704 39118067

[39] Jin X, Xu Q, Pu C, Zhu K, Lu C, Jiang Y, et al. Therapeutic efficacy of anti-CD19 CAR-T cells in a mouse model of systemic lupus erythematosus. Cell Mol Immunol. 2021;18(8):1896–903. 32472023 10.1038/s41423-020-0472-1 PMC8322088

[40] Kansal R, Richardson N, Neeli I, Khawaja S, Chamberlain D, Ghani M et al. Sustained B cell depletion by CD19-targeted CAR T cells is a highly effective treatment for murine lupus. Sci Transl Med. 2019;11(482). 10.1126/scitranslmed.aav1648 PMC8201923 30842314

[41] Mougiakakos D, Krönke G, Völkl S, Kretschmann S, Aigner M, Kharboutli S, et al. CD19-Targeted CAR T cells in refractory systemic Lupus Erythematosus. N Engl J Med. 2021;385(6):567–9. 34347960 10.1056/NEJMc2107725

[42] Mackensen A, Müller F, Mougiakakos D, Böltz S, Wilhelm A, Aigner M, et al. Anti-CD19 CAR T cell therapy for refractory systemic lupus erythematosus. Nat Med. 2022;28(10):2124–32. 36109639 10.1038/s41591-022-02017-5

[43] Taubmann J, Müller F, Yalcin Mutlu M, Völkl S, Aigner M, Bozec A et al. CD19 Chimeric Antigen Receptor T Cell Treatment: unraveling the role of B cells in systemic Lupus Erythematosus. Arthritis & rheumatology (Hoboken, NJ). 2024;76(4):497–504. 10.1002/art.42784 38114423

[44] Feng J, Hu Y, Chang A, Huang H. CD19/BCMA CAR-T cell therapy for refractory systemic lupus erythematosus - safety and preliminary Efficacy Data from a phase I clinical study. Blood. 2023;142:4835.

[45] Zhang W, Feng J, Cinquina A, Wang Q, Xu H, Zhang Q, et al. Treatment of systemic lupus erythematosus using BCMA-CD19 compound CAR. Stem cell Reviews Rep. 2021;17(6):2120–3. 10.1007/s12015-021-10251-6 PMC8599262 34458965

[46] Wang W, He S, Zhang W, Zhang H, DeStefano VM, Wada M, et al. BCMA-CD19 compound CAR T cells for systemic lupus erythematosus: a phase 1 open-label clinical trial. Ann Rheum Dis. 2024;83(10):1304–14. 38777376 10.1136/ard-2024-225785

[47] Doglio M, Ugolini A, Bercher-Brayer C, Camisa B, Toma C, Norata R, et al. Regulatory T cells expressing CD19-targeted chimeric antigen receptor restore homeostasis in systemic Lupus Erythematosus. Nat Commun. 2024;15(1):2542. 38538608 10.1038/s41467-024-46448-9 PMC10973480

[48] McInnes IB, Schett G. Pathogenetic insights from the treatment of rheumatoid arthritis. Lancet (London England). 2017;389(10086):2328–37. 28612747 10.1016/S0140-6736(17)31472-1

[49] Association CR. 2018 Chinese guideline for the diagnosis and treatment of rheumatoid arthritis. Clin Res Pract. 2018;3(12):201.

[50] Zhang B, Wang Y, Yuan Y, Sun J, Liu L, Huang D, et al. In vitro elimination of autoreactive B cells from rheumatoid arthritis patients by universal chimeric antigen receptor T cells. Ann Rheum Dis. 2021;80(2):176–84. 32998865 10.1136/annrheumdis-2020-217844

[51] Bao L, Bo XC, Cao HW, Qian C, Wang Z, Li B. Engineered T cells and their therapeutic applications in autoimmune diseases. Zoological Res. 2022;43(2):150–65. 10.24272/j.issn.2095-8137.2021.363 PMC8920845 35008131

[52] Haghikia A, Hegelmaier T, Wolleschak D, Böttcher M, Pappa V, Motte J et al. Clinical efficacy and autoantibody seroconversion with CD19-CAR T cell therapy in a patient with rheumatoid arthritis and coexisting myasthenia gravis. Annals of the rheumatic diseases. 2024. 10.1136/ard-2024-226017 38937071

[53] Raffin C, Muller Y, Barragan J, Zhou Y, Piccoli L, Lanzavecchia A, et al. Development of citrullinated-vimentin-specific CAR for targeting Tregs to treat autoimmune rheumatoid arthritis. J Immunol. 2019;202(1Supplement):1332–2.

[54] Liu L. Research progress on the the function of cytokines in Sjogren’s syndrome. J Qiqihar Med Univ. 2020;41(22):2855–9.

[55] Jin L, Dai M, Li C, Wang J, Wu B. Risk factors for primary Sjögren’s syndrome: a systematic review and meta-analysis. Clin Rheumatol. 2023;42(2):327–38. 36534351 10.1007/s10067-022-06474-8 PMC9873717

[56] Bjordal O, Norheim KB, Rødahl E, Jonsson R, Omdal R. Primary Sjögren’s syndrome and the eye. Surv Ophthalmol. 2020;65(2):119–32. 31634487 10.1016/j.survophthal.2019.10.004

[57] Qin B, Wang J, Yang Z, Yang M, Ma N, Huang F, et al. Epidemiology of primary Sjögren’s syndrome: a systematic review and meta-analysis. Ann Rheum Dis. 2015;74(11):1983–9. 24938285 10.1136/annrheumdis-2014-205375

[58] Meng H, Sun X, Song Y, Zou J, An G, Jin Z, et al. La/SSB chimeric autoantibody receptor modified NK92MI cells for targeted therapy of autoimmune disease. Clin Immunol (Orlando Fla). 2018;192:40–9. 10.1016/j.clim.2018.04.006 29673902

[59] Sheng L, Zhang Y, Song Q, Jiang X, Cao W, Li L, et al. Concurrent remission of lymphoma and Sjögren’s disease following anti-CD19 chimeric antigen receptor-T cell therapy for diffuse large B-cell lymphoma: a case report. Front Immunol. 2023;14:1298815. 38173731 10.3389/fimmu.2023.1298815 PMC10762793

[60] Thurgood LA, Arentz G, Lindop R, Jackson MW, Whyte AF, Colella AD, et al. An immunodominant La/SSB autoantibody proteome derives from public clonotypes. Clin Exp Immunol. 2013;174(2):237–44. 23841690 10.1111/cei.12171 PMC3828827

[61] Tian DC, Zhang C, Yuan M, Yang X, Gu H, Li Z, et al. Incidence of multiple sclerosis in China: a nationwide hospital-based study. Lancet Reg Health Western Pac. 2020;1:100010. 10.1016/j.lanwpc.2020.100010 PMC8315658 34327341

[62] McFarland HF, Martin R. Multiple sclerosis: a complicated picture of autoimmunity. Nat Immunol. 2007;8(9):913–9. 17712344 10.1038/ni1507

[63] Neuroimmunology CSo, Fu-Dong S. Chinese guidelines for diagnosis and treatment of multiple sclerosis (2023 edition). Chin J Neurol. 2024(01):10–23.

[64] Magliozzi R, Howell O, Vora A, Serafini B, Nicholas R, Puopolo M, et al. Meningeal B-cell follicles in secondary progressive multiple sclerosis associate with early onset of disease and severe cortical pathology. Brain. 2007;130(Pt 4):1089–104. 17438020 10.1093/brain/awm038

[65] Mitsdoerffer M, Di Liberto G, Dötsch S, Sie C, Wagner I, Pfaller M, et al. Formation and immunomodulatory function of meningeal B cell aggregates in progressive CNS autoimmunity. Brain. 2021;144(6):1697–710. 33693558 10.1093/brain/awab093

[66] Gupta S, Simic M, Sagan SA, Shepherd C, Duecker J, Sobel RA et al. CAR-T cell-mediated B-Cell depletion in Central Nervous System Autoimmunity. Neurology(R) Neuroimmunol Neuroinflammation. 2023;10(2). 10.1212/NXI.0000000000200080 PMC9853314 36657993

[67] Fischbach F, Richter J, Pfeffer LK, Fehse B, Berger SC, Reinhardt S, et al. CD19-targeted chimeric antigen receptor T cell therapy in two patients with multiple sclerosis. Med (New York NY). 2024;5(6):550–e82. 10.1016/j.medj.2024.03.002 38554710

[68] De Paula Pohl A, Schmidt A, Zhang AH, Maldonado T, Königs C, Scott DW. Engineered regulatory T cells expressing myelin-specific chimeric antigen receptors suppress EAE progression. Cell Immunol. 2020;358:104222. 33053469 10.1016/j.cellimm.2020.104222

[69] Fransson M, Piras E, Burman J, Nilsson B, Essand M, Lu B, et al. CAR/FoxP3-engineered T regulatory cells target the CNS and suppress EAE upon intranasal delivery. J Neuroinflamm. 2012;9:112. 10.1186/1742-2094-9-112 PMC3403996 22647574

[70] Kim YC, Zhang AH, Yoon J, Culp WE, Lees JR, Wucherpfennig KW, et al. Engineered MBP-specific human tregs ameliorate MOG-induced EAE through IL-2-triggered inhibition of effector T cells. J Autoimmun. 2018;92:77–86. 29857928 10.1016/j.jaut.2018.05.003 PMC6054915

[71] Chang T. Chinese guidelines for the diagnosis and treatment of Myasthenia gravis (2020 Edition). Chin J Neuroimmunol Neurol. 2021;28(01):1–12.

[72] Kexin J, Chongbo Z. Advancements in the understanding and diagnostic techniques of pathogenic autoantibodies in serum of patients with myasthenia gravis. Chin J Neurol. 2024;57(01):5–9.

[73] Oh S, Mao X, Manfredo-Vieira S, Lee J, Patel D, Choi EJ, et al. Precision targeting of autoantigen-specific B cells in muscle-specific tyrosine kinase myasthenia gravis with chimeric autoantibody receptor T cells. Nat Biotechnol. 2023;41(9):1229–38. 36658341 10.1038/s41587-022-01637-z PMC10354218

[74] Haghikia A, Hegelmaier T, Wolleschak D, Böttcher M, Desel C, Borie D, et al. Anti-CD19 CAR T cells for refractory myasthenia gravis. Lancet Neurol. 2023;22(12):1104–5. 37977704 10.1016/S1474-4422(23)00375-7

[75] Granit V, Benatar M, Kurtoglu M, Miljković MD, Chahin N, Sahagian G, et al. Safety and clinical activity of autologous RNA chimeric antigen receptor T-cell therapy in myasthenia gravis (MG-001): a prospective, multicentre, open-label, non-randomised phase 1b/2a study. Lancet Neurol. 2023;22(7):578–90. 37353278 10.1016/S1474-4422(23)00194-1 PMC10416207

[76] Tian DS, Qin C, Dong MH, Heming M, Zhou LQ, Wang W, et al. B cell lineage reconstitution underlies CAR-T cell therapeutic efficacy in patients with refractory myasthenia gravis. EMBO Mol Med. 2024;16(4):966–87. 38409527 10.1038/s44321-024-00043-z PMC11018773

[77] Wingerchuk DM, Banwell B, Bennett JL, Cabre P, Carroll W, Chitnis T, et al. International consensus diagnostic criteria for neuromyelitis optica spectrum disorders. Neurology. 2015;85(2):177–89. 26092914 10.1212/WNL.0000000000001729 PMC4515040

[78] Qin C, Tian DS, Zhou LQ, Shang K, Huang L, Dong MH, et al. Anti-BCMA CAR T-cell therapy CT103A in relapsed or refractory AQP4-IgG seropositive neuromyelitis optica spectrum disorders: phase 1 trial interim results. Signal Transduct Target Therapy. 2023;8(1):5. 10.1038/s41392-022-01278-3 PMC9810610 36596762

[79] Lundberg IE, Fujimoto M, Vencovsky J, Aggarwal R, Holmqvist M, Christopher-Stine L, et al. Idiopathic inflammatory myopathies. Nat Reviews Disease Primers. 2021;7(1):86. 34857798 10.1038/s41572-021-00321-x

[80] Pu C. Idiopathic inflammatory myopathy. Chin J Neurol. 2019(05):410–22.

[81] Pecher AC, Hensen L, Klein R, Schairer R, Lutz K, Atar D, et al. CD19-Targeting CAR T cells for myositis and interstitial lung Disease Associated with antisynthetase syndrome. JAMA. 2023;329(24):2154–62. 37367976 10.1001/jama.2023.8753 PMC10300719

[82] Müller F, Boeltz S, Knitza J, Aigner M, Völkl S, Kharboutli S, et al. CD19-targeted CAR T cells in refractory antisynthetase syndrome. Lancet (London England). 2023;401(10379):815–8. 36930673 10.1016/S0140-6736(23)00023-5

[83] Taubmann J, Knitza J, Müller F, Völkl S, Aigner M, Kleyer A, et al. Rescue therapy of antisynthetase syndrome with CD19-targeted CAR-T cells after failure of several B-cell depleting antibodies. Rheumatology (Oxford). 2024;63(1):e12–4. 37432378 10.1093/rheumatology/kead330 PMC10765150

[84] Wang X, Wu X, Tan B, Zhu L, Zhang Y, Lin L, et al. Allogeneic CD19-targeted CAR-T therapy in patients with severe myositis and systemic sclerosis. Cell. 2024;187(18):4890–e9049. 39013470 10.1016/j.cell.2024.06.027

[85] Diagnosis. and Treatment of pemphigus vulgaris: an expert proposal (202 0). Chin J Dermatol.53(1).

[86] Zhang Y, Niu X. Update of targeted treatment of pemphigus. China J Lepr Skin Dis. 2024;40(03):221–4.

[87] Ellebrecht CT, Bhoj VG, Nace A, Choi EJ, Mao X, Cho MJ, et al. Reengineering chimeric antigen receptor T cells for targeted therapy of autoimmune disease. Sci (New York NY). 2016;353(6295):179–84. 10.1126/science.aaf6756 PMC5343513 27365313

[88] Lee J, Lundgren DK, Mao X, Manfredo-Vieira S, Nunez-Cruz S, Williams EF, et al. Antigen-specific B cell depletion for precision therapy of mucosal pemphigus vulgaris. J Clin Investig. 2020;130(12):6317–24. 32817591 10.1172/JCI138416 PMC7685721

[89] Taïeb A, Picardo M. The definition and assessment of vitiligo: a consensus report of the Vitiligo European Task Force. Pigment Cell Res. 2007;20(1):27–35. 17250545 10.1111/j.1600-0749.2006.00355.x

[90] Ezzedine K, Eleftheriadou V, Whitton M, van Geel N, Vitiligo. Lancet (London England). 2015;386(9988):74–84. 10.1016/S0140-6736(14)60763-7 25596811

[91] Le Poole IC, van den Wijngaard RM, Westerhof W, Dutrieux RP, Das PK. Presence or absence of melanocytes in vitiligo lesions: an immunohistochemical investigation. J Invest Dermatol. 1993;100(6):816–22. 7684427 10.1111/1523-1747.ep12476645

[92] Klarquist J, Denman CJ, Hernandez C, Wainwright DA, Strickland FM, Overbeck A, et al. Reduced skin homing by functional Treg in Vitiligo. Pigment cell Melanoma Res. 2010;23(2):276–86. 20175879 10.1111/j.1755-148X.2010.00688.x PMC3778930

[93] Le Poole IC, Mehrotra S. Replenishing Regulatory T Cells to Halt Depigmentation in Vitiligo. J Invest Dermatology Symp Proc. 2017;18(2):S38–45. 10.1016/j.jisp.2016.10.023 28941492

[94] Chatterjee S, Eby JM, Al-Khami AA, Soloshchenko M, Kang HK, Kaur N, et al. A quantitative increase in regulatory T cells controls development of vitiligo. J Invest Dermatol. 2014;134(5):1285–94. 24366614 10.1038/jid.2013.540 PMC3989443

[95] Le Poole IC, Stennett LS, Bonish BK, Dee L, Robinson JK, Hernandez C, et al. Expansion of vitiligo lesions is associated with reduced epidermal CDw60 expression and increased expression of HLA-DR in perilesional skin. Br J Dermatol. 2003;149(4):739–48. 14616364 10.1046/j.1365-2133.2003.05539.x

[96] Mukhatayev Z, Dellacecca ER, Cosgrove C, Shivde R, Jaishankar D, Pontarolo-Maag K, et al. Antigen specificity enhances Disease Control by Tregs in Vitiligo. Front Immunol. 2020;11:581433. 33335528 10.3389/fimmu.2020.581433 PMC7736409

[97] He B, Li X, Zhou Z. Chinese guidelines for the diagnosis and treatment of type 1 diabetes Mellitus. Edition). 2021;2022(1):1123–7.

[98] Zhang L, Sosinowski T, Cox AR, Cepeda JR, Sekhar NS, Hartig SM, et al. Chimeric antigen receptor (CAR) T cells targeting a pathogenic MHC class II:peptide complex modulate the progression of autoimmune diabetes. J Autoimmun. 2019;96:50–8. 30122420 10.1016/j.jaut.2018.08.004 PMC6541442

[99] Tenspolde M, Zimmermann K, Weber LC, Hapke M, Lieber M, Dywicki J, et al. Regulatory T cells engineered with a novel insulin-specific chimeric antigen receptor as a candidate immunotherapy for type 1 diabetes. J Autoimmun. 2019;103:102289. 31176558 10.1016/j.jaut.2019.05.017

[100] Radichev IA, Yoon J, Scott DW, Griffin K, Savinov AY. Towards antigen-specific Tregs for type 1 diabetes: construction and functional assessment of pancreatic endocrine marker, HPi2-based chimeric antigen receptor. Cell Immunol. 2020;358:104224. 33068914 10.1016/j.cellimm.2020.104224 PMC7655659

[101] Imam S, Jaume J. MON-LB033 unleashing the anti-inflammatory potential of Treg Cells against Type I diabetes using advanced chimeric Antigen receptor technology. J Endocr Soc. 2019;3(Supplement_1).

[102] Bruner LP, White AM, Proksell S. Inflamm Bowel Disease Prim care. 2023;50(3):411–27. 10.1016/j.pop.2023.03.009 37516511

[103] Elinav E, Waks T, Eshhar Z. Redirection of regulatory T cells with predetermined specificity for the treatment of experimental colitis in mice. Gastroenterology. 2008;134(7):2014–24. 18424268 10.1053/j.gastro.2008.02.060

[104] Blat D, Zigmond E, Alteber Z, Waks T, Eshhar Z. Suppression of murine colitis and its associated cancer by carcinoembryonic antigen-specific regulatory T cells. Mol Therapy: J Am Soc Gene Therapy. 2014;22(5):1018–28. 10.1038/mt.2014.41 PMC4015241 24686242

[105] Boardman DA, Wong MQ, Rees WD, Wu D, Himmel ME, Orban PC, et al. Flagellin-specific human CAR tregs for immune regulation in IBD. J Autoimmun. 2023;134:102961. 36470208 10.1016/j.jaut.2022.102961 PMC9908852

[106] Hammad H, Lambrecht BN. The basic immunology of asthma. Cell. 2021;184(9):2521–2. 33930297 10.1016/j.cell.2021.04.019

[107] Jin G, Liu Y, Wang L, He Z, Zhao X, Ma Y et al. A single infusion of engineered long-lived and multifunctional T cells confers durable remission of asthma in mice. Nat Immunol. 2024. 10.1038/s41590-024-01834-9 38802511

[108] Shamsuddin SH, Jayne DG, Tomlinson DC, McPherson MJ, Millner PA. Selection and characterisation of Affimers specific for CEA recognition. Sci Rep. 2021;11(1):744. 33436840 10.1038/s41598-020-80354-6 PMC7804248

[109] Skuljec J, Chmielewski M, Happle C, Habener A, Busse M, Abken H, et al. Chimeric Antigen Receptor-Redirected Regulatory T Cells Suppress Experimental Allergic Airway Inflammation, a model of Asthma. Front Immunol. 2017;8:1125. 28955341 10.3389/fimmu.2017.01125 PMC5600908

[110] Schett G, Müller F, Taubmann J, Mackensen A, Wang W, Furie RA, et al. Advancements and challenges in CAR T cell therapy in autoimmune diseases. Nat Rev Rheumatol. 2024;20(9):531–44. 39107407 10.1038/s41584-024-01139-z

[111] Müller F, Taubmann J, Bucci L, Wilhelm A, Bergmann C, Völkl S, et al. CD19 CAR T-Cell therapy in Autoimmune Disease - A Case Series with Follow-up. N Engl J Med. 2024;390(8):687–700. 38381673 10.1056/NEJMoa2308917

[112] Qin C, Dong MH, Zhou LQ, Wang W, Cai SB, You YF, et al. Single-cell analysis of refractory anti-SRP necrotizing myopathy treated with anti-BCMA CAR-T cell therapy. Proc Natl Acad Sci USA. 2024;121(6):e2315990121. 38289960 10.1073/pnas.2315990121 PMC10861907

[113] AIDS and Hepatitis C Professional Group SoID, Chinese Medical Association, Prevention CCfDCa. Chinese guidelines for diagnosis and treatment of HIV/AIDS. (2021 edition). Chinese Journal of Clinical Infectious Diseases. 2021.

[114] Seif M, Einsele H, Löffler J. CAR T cells beyond Cancer: hope for Immunomodulatory Therapy of Infectious diseases. Front Immunol. 2019;10:2711. 31824500 10.3389/fimmu.2019.02711 PMC6881243

[115] Mitsuyasu RT, Anton PA, Deeks SG, Scadden DT, Connick E, Downs MT, et al. Prolonged survival and tissue trafficking following adoptive transfer of CD4zeta gene-modified autologous CD4(+) and CD8(+) T cells in human immunodeficiency virus-infected subjects. Blood. 2000;96(3):785–93. 10910888

[116] Deeks SG, Wagner B, Anton PA, Mitsuyasu RT, Scadden DT, Huang C, et al. A phase II randomized study of HIV-specific T-cell gene therapy in subjects with undetectable plasma viremia on combination antiretroviral therapy. Mol Therapy: J Am Soc Gene Therapy. 2002;5(6):788–97. 10.1006/mthe.2002.0611 12027564

[117] Scholler J, Brady TL, Binder-Scholl G, Hwang WT, Plesa G, Hege KM, et al. Decade-long safety and function of retroviral-modified chimeric antigen receptor T cells. Sci Transl Med. 2012;4(132):132ra53. 22553251 10.1126/scitranslmed.3003761 PMC4368443

[118] Tebas P, Stein D, Tang WW, Frank I, Wang SQ, Lee G, et al. Gene editing of CCR5 in autologous CD4 T cells of persons infected with HIV. N Engl J Med. 2014;370(10):901–10. 24597865 10.1056/NEJMoa1300662 PMC4084652

[119] Tebas P, Jadlowsky JK, Shaw PA, Tian L, Esparza E, Brennan AL et al. CCR5-edited CD4 + T cells augment HIV-specific immunity to enable post-rebound control of HIV replication. J Clin Investig. 2024;134(9). 10.1172/JCI181576 PMC11060720 38690741

[120] Xu L, Wang J, Liu Y, Xie L, Su B, Mou D, et al. CRISPR-Edited stem cells in a patient with HIV and Acute lymphocytic leukemia. N Engl J Med. 2019;381(13):1240–7. 31509667 10.1056/NEJMoa1817426

[121] Hale M, Mesojednik T, Romano Ibarra GS, Sahni J, Bernard A, Sommer K, et al. Engineering HIV-Resistant, Anti-HIV Chimeric Antigen Receptor T Cells. Mol Therapy: J Am Soc Gene Therapy. 2017;25(3):570–9. 10.1016/j.ymthe.2016.12.023 PMC5363191 28143740

[122] Matsui Y, Miura Y. Advancements in cell-based therapies for HIV Cure. Cells. 2023;13(1). 10.3390/cells13010064 PMC10778010 38201268

[123] Gaudinski MR, Houser KV, Doria-Rose NA, Chen GL, Rothwell RSS, Berkowitz N, et al. Safety and pharmacokinetics of broadly neutralising human monoclonal antibody VRC07-523LS in healthy adults: a phase 1 dose-escalation clinical trial. Lancet HIV. 2019;6(10):e667–79. 31473167 10.1016/S2352-3018(19)30181-X PMC11100866

[124] Campos-Gonzalez G, Martinez-Picado J, Velasco-Hernandez T, Salgado M. Opportunities for CAR-T cell immunotherapy in HIV Cure. Viruses. 2023;15(3). 10.3390/v15030789 PMC10057306 36992496

[125] Mao Y, Liao Q, Zhu Y, Bi M, Zou J, Zheng N, et al. Efficacy and safety of novel multifunctional M10 CAR-T cells in HIV-1-infected patients: a phase I, multicenter, single-arm, open-label study. Cell Discovery. 2024;10(1):49. 38740803 10.1038/s41421-024-00658-z PMC11091177

[126] Liu B, Zhang W, Xia B, Jing S, Du Y, Zou F et al. Broadly neutralizing antibody-derived CAR T cells reduce viral reservoir in individuals infected with HIV-1. J Clin Investig. 2021;131(19). 10.1172/JCI150211 PMC8483761 34375315

[127] Hemelaar J. The origin and diversity of the HIV-1 pandemic. Trends Mol Med. 2012;18(3):182–92. 22240486 10.1016/j.molmed.2011.12.001

[128] Perreau M, Savoye A-L, De Crignis E, Corpataux J-M, Cubas R, Haddad EK, et al. Follicular helper T cells serve as the major CD4 T cell compartment for HIV-1 infection, replication, and production. J Exp Med. 2012;210(1):143–56. 23254284 10.1084/jem.20121932 PMC3549706

[129] Eichholz K, Fukazawa Y, Peterson CW, Haeseleer F, Medina M, Hoffmeister S et al. Anti-PD-1 chimeric antigen receptor T cells efficiently target SIV-infected CD4 + T cells in germinal centers. J Clin Investig. 2024;134(7). 10.1172/JCI169309 PMC10977982 38557496

[130] Liu L, Patel B, Ghanem MH, Bundoc V, Zheng Z, Morgan RA, et al. Novel CD4-Based bispecific chimeric Antigen receptor designed for enhanced Anti-HIV potency and absence of HIV Entry receptor activity. J Virol. 2015;89(13):6685–94. 25878112 10.1128/JVI.00474-15 PMC4468509

[131] Ghanem MH, Bolivar-Wagers S, Dey B, Hajduczki A, Vargas-Inchaustegui DA, Danielson DT, et al. Bispecific chimeric antigen receptors targeting the CD4 binding site and high-mannose glycans of gp120 optimized for anti-human immunodeficiency virus potency and breadth with minimal immunogenicity. Cytotherapy. 2018;20(3):407–19. 29306566 10.1016/j.jcyt.2017.11.001

[132] Maldini CR, Ellis GI, Riley JL. CAR T cells for infection, autoimmunity and allotransplantation. Nat Rev Immunol. 2018;18(10):605–16. 30046149 10.1038/s41577-018-0042-2 PMC6505691

[133] Hajduczki A, Danielson DT, Elias DS, Bundoc V, Scanlan AW, Berger EA. A trispecific Anti-HIV chimeric Antigen receptor containing the CCR5 N-Terminal region. Front Cell Infect Microbiol. 2020;10. 10.3389/fcimb.2020.00242 PMC7261873 32523897

[134] El-Mowafy M, El-Mesery M, Khalil MAF, El-Mesery A, Elgaml A. Expression and purification of Hepatitis B virus core antigen using Escherichia coli and its utilization for the diagnosis of Hepatitis B virus infections. Biologicals. 2024;85:101726. 37979341 10.1016/j.biologicals.2023.101726

[135] Bohne F, Chmielewski M, Ebert G, Wiegmann K, Kürschner T, Schulze A, et al. T cells redirected against hepatitis B virus surface proteins eliminate infected hepatocytes. Gastroenterology. 2008;134(1):239–47. 18166356 10.1053/j.gastro.2007.11.002

[136] Krebs K, Böttinger N, Huang LR, Chmielewski M, Arzberger S, Gasteiger G, et al. T cells expressing a chimeric antigen receptor that binds hepatitis B virus envelope proteins control virus replication in mice. Gastroenterology. 2013;145(2):456–65. 23639914 10.1053/j.gastro.2013.04.047

[137] Kruse RL, Shum T, Tashiro H, Barzi M, Yi Z, Whitten-Bauer C, et al. HBsAg-redirected T cells exhibit antiviral activity in HBV-infected human liver chimeric mice. Cytotherapy. 2018;20(5):697–705. 29631939 10.1016/j.jcyt.2018.02.002 PMC6038120

[138] Klopp A, Schreiber S, Kosinska AD, Pulé M, Protzer U, Wisskirchen K. Depletion of T cells via Inducible Caspase 9 increases safety of adoptive T-Cell therapy against chronic Hepatitis B. Front Immunol. 2021;12:734246. 34691041 10.3389/fimmu.2021.734246 PMC8527178

[139] Xiong Y, Zhang C, Wang X. Hepatitis C Virus genomic structure and function. Chin J Biochem Mol Biology. 2008;07:587–92.

[140] Sautto GA, Wisskirchen K, Clementi N, Castelli M, Diotti RA, Graf J, et al. Chimeric antigen receptor (CAR)-engineered T cells redirected against hepatitis C virus (HCV) E2 glycoprotein. Gut. 2016;65(3):512–23. 25661083 10.1136/gutjnl-2014-308316 PMC4789830

[141] Zhang A, Williamson CD, Wong DS, Bullough MD, Brown KJ, Hathout Y et al. Quantitative proteomic analyses of Human Cytomegalovirus-Induced Restructuring of endoplasmic reticulum-mitochondrial contacts at late times of infection *. Mol Cell Proteom. 2011;10(10). 10.1074/mcp.M111.009936 PMC3205871 21742798

[142] Weekes MP, Tomasec P, Huttlin EL, Fielding CA, Nusinow D, Stanton RJ, et al. Quantitative temporal viromics: an approach to investigate host-pathogen interaction. Cell. 2014;157(6):1460–72. 24906157 10.1016/j.cell.2014.04.028 PMC4048463

[143] Olbrich H, Theobald SJ, Slabik C, Gerasch L, Schneider A, Mach M, et al. Adult and Cord Blood-Derived High-Affinity gB-CAR-T cells effectively react against human cytomegalovirus infections. Hum Gene Ther. 2020;31(7–8):423–39. 32159399 10.1089/hum.2019.149 PMC7194322

[144] Proff J, Walterskirchen C, Brey C, Geyeregger R, Full F, Ensser A, et al. Cytomegalovirus-infected cells resist T cell mediated killing in an HLA-Recognition independent manner. Front Microbiol. 2016;7:844. 27375569 10.3389/fmicb.2016.00844 PMC4899442

[145] Zhu Q-Y, Shan S, Yu J, Peng S-Y, Sun C, Zuo Y, et al. A potent and protective human neutralizing antibody targeting a novel vulnerable site of Epstein-Barr virus. Nat Commun. 2021;12(1):6624. 34785638 10.1038/s41467-021-26912-6 PMC8595662

[146] Cui X, Snapper CM. Epstein Barr Virus: development of vaccines and Immune Cell Therapy for EBV-Associated diseases. Front Immunol. 2021;12:734471. 34691042 10.3389/fimmu.2021.734471 PMC8532523

[147] Tang X, Zhou Y, Li W, Tang Q, Chen R, Zhu J, et al. T cells expressing a LMP1-specific chimeric antigen receptor mediate antitumor effects against LMP1-positive nasopharyngeal carcinoma cells in vitro and in vivo. J Biomedical Res. 2014;28(6):468–75. 10.7555/JBR.28.20140066 PMC4250525 25469116

[148] Slabik C, Kalbarczyk M, Danisch S, Zeidler R, Klawonn F, Volk V, et al. CAR-T cells targeting Epstein-Barr Virus gp350 validated in a Humanized Mouse Model of EBV infection and lymphoproliferative disease. Mol Therapy Oncolytics. 2020;18:504–24. 10.1016/j.omto.2020.08.005 PMC7479496 32953984

[149] Rodriguez-Morales AJ, Bonilla-Aldana DK, Balbin-Ramon GJ, Rabaan AA, Sah R, Paniz-Mondolfi A, et al. History is repeating itself: probable zoonotic spillover as the cause of the 2019 novel Coronavirus Epidemic. Le Infezioni in Medicina. 2020;28(1):3–5. 32009128

[150] Yadav R, Chaudhary JK, Jain N, Chaudhary PK, Khanra S, Dhamija P et al. Role of structural and non-structural proteins and therapeutic targets of SARS-CoV-2 for COVID-19. Cells. 2021;10(4). 10.3390/cells10040821 PMC8067447 33917481

[151] Ma M, Badeti S, Geng K, Liu D. Efficacy of Targeting SARS-CoV-2 by CAR-NK Cells. bioRxiv: the preprint server for biology. 2020.

[152] Christodoulou I, Rahnama R, Ravich JW, Seo J, Zolov SN, Marple AN, et al. Glycoprotein targeted CAR-NK cells for the treatment of SARS-CoV-2 infection. Front Immunol. 2021;12:763460. 35003077 10.3389/fimmu.2021.763460 PMC8732772

[153] Seif M, Kakoschke TK, Ebel F, Bellet MM, Trinks N, Renga G, et al. CAR T cells targeting aspergillus fumigatus are effective at treating invasive pulmonary aspergillosis in preclinical models. Sci Transl Med. 2022;14(664):eabh1209. 36170447 10.1126/scitranslmed.abh1209

[154] Brown GD. Dectin-1: a signalling non-TLR pattern-recognition receptor. Nat Rev Immunol. 2006;6(1):33–43. 16341139 10.1038/nri1745

[155] Kumaresan PR, Manuri PR, Albert ND, Maiti S, Singh H, Mi T, et al. Bioengineering T cells to target carbohydrate to treat opportunistic fungal infection. Proc Natl Acad Sci USA. 2014;111(29):10660–5. 25002471 10.1073/pnas.1312789111 PMC4115509

[156] O’Meara TR, Alspaugh JA. The Cryptococcus neoformans capsule: a sword and a shield. Clin Microbiol Rev. 2012;25(3):387–408. 22763631 10.1128/CMR.00001-12 PMC3416491

[157] da Silva TA, Hauser PJ, Bandey I, Laskowski T, Wang Q, Najjar AM, et al. Glucuronoxylomannan in the Cryptococcus species capsule as a target for chimeric Antigen receptor T-cell therapy. Cytotherapy. 2021;23(2):119–30. 33303326 10.1016/j.jcyt.2020.11.002 PMC11375790

[158] Parida SK, Poiret T, Zhenjiang L, Meng Q, Heyckendorf J, Lange C, et al. T-Cell therapy: options for infectious diseases. Clin Infect Diseases: Official Publication Infect Dis Soc Am. 2015;61Suppl(3):S217–24. 10.1093/cid/civ615 PMC4583575 26409284

[159] Henderson NC, Rieder F, Wynn TA. Fibrosis: from mechanisms to medicines. Nature. 2020;587(7835):555–66. 33239795 10.1038/s41586-020-2938-9 PMC8034822

[160] Zebrowski DC, Vergarajauregui S, Wu CC, Piatkowski T, Becker R, Leone M et al. Developmental alterations in centrosome integrity contribute to the post-mitotic state of mammalian cardiomyocytes. eLife. 2015;4. 10.7554/eLife.05563 PMC4541494 26247711

[161] Zeng M, Wang H. Current status and perspectives on the diagnosis and therapeutics of myocardialfibrosis. Chin J Front Med. 2023;15(03):64–71.

[162] Aghajanian H, Kimura T, Rurik JG, Hancock AS, Leibowitz MS, Li L, et al. Targeting cardiac fibrosis with engineered T cells. Nature. 2019;573(7774):430–3. 31511695 10.1038/s41586-019-1546-z PMC6752964

[163] Rurik JG, Tombácz I, Yadegari A, Méndez Fernández PO, Shewale SV, Li L, et al. CAR T cells produced in vivo to treat cardiac injury. Sci (New York NY). 2022;375(6576):91–6. 10.1126/science.abm0594 PMC9983611 34990237

[164] Lu L, You H, Xie W, Jia J. Consensus on the diagnosis and therapy of hepaticfibrosis. J Practical Hepatol. 2019;22(06):793–803.

[165] Masucci MT, Minopoli M, Di Carluccio G, Motti ML, Carriero MV. Therapeutic strategies targeting urokinase and its receptor in Cancer. Cancers. 2022;14(3). 10.3390/cancers14030498 PMC8833673 35158766

[166] Schuliga M, Jaffar J, Harris T, Knight DA, Westall G, Stewart AG. The fibrogenic actions of lung fibroblast-derived urokinase: a potential drug target in IPF. Sci Rep. 2017;7:41770. 28139758 10.1038/srep41770 PMC5282574

[167] Amor C, Feucht J, Leibold J, Ho YJ, Zhu C, Alonso-Curbelo D, et al. Senolytic CAR T cells reverse senescence-associated pathologies. Nature. 2020;583(7814):127–32. 32555459 10.1038/s41586-020-2403-9 PMC7583560

[168] Dai H, Zhu C, Huai Q, Xu W, Zhu J, Zhang X, et al. Chimeric antigen receptor-modified macrophages ameliorate liver fibrosis in preclinical models. J Hepatol. 2024;80(6):913–27. 38340812 10.1016/j.jhep.2024.01.034

[169] Gao J, Wang Y, Meng X, Wang X, Han F, Xing H, et al. A FAPα-activated MRI nanoprobe for precise grading diagnosis of clinical liver fibrosis. Nat Commun. 2024;15(1):8036. 39271701 10.1038/s41467-024-52308-3 PMC11399433

[170] Tacke F, Puengel T, Loomba R, Friedman SL. An integrated view of anti-inflammatory and antifibrotic targets for the treatment of NASH. J Hepatol. 2023;79(2):552–66. 37061196 10.1016/j.jhep.2023.03.038 PMC12164378

[171] Shahvali S, Rahiman N, Jaafari MR, Arabi L. Targeting fibroblast activation protein (FAP): advances in CAR-T cell, antibody, and vaccine in cancer immunotherapy. Drug Delivery Translational Res. 2023;13(7):2041–56. 10.1007/s13346-023-01308-9 36840906

[172] Ferrer-Curriu G, Soler-Botija C, Charvatova S, Motais B, Roura S, Galvez-Monton C, et al. Preclinical scenario of targeting myocardial fibrosis with chimeric antigen receptor (CAR) immunotherapy. Volume 158. Biomedicine & pharmacotherapy = Biomedecine & pharmacotherapie; 2023. p. 114061. 10.1016/j.biopha.2022.114061 36495661

[173] Renault NK, Dyack S, Dobson MJ, Costa T, Lam WL, Greer WL. Heritable skewed X-chromosome inactivation leads to haemophilia A expression in heterozygous females. Eur J Hum Genetics: EJHG. 2007;15(6):628–37. 17342157 10.1038/sj.ejhg.5201799

[174] Parvathaneni K, Scott DW. Engineered FVIII-expressing cytotoxic T cells target and kill FVIII-specific B cells in vitro and in vivo. Blood Adv. 2018;2(18):2332–40. 30232086 10.1182/bloodadvances.2018018556 PMC6156881

[175] Yoon J, Schmidt A, Zhang AH, Königs C, Kim YC, Scott DW. FVIII-specific human chimeric antigen receptor T-regulatory cells suppress T- and B-cell responses to FVIII. Blood. 2017;129(2):238–45. 28064157 10.1182/blood-2016-07-727834 PMC5234219

[176] Fu RY, Chen AC, Lyle MJ, Chen CY, Liu CL, Miao CH. CD4(+) T cells engineered with FVIII-CAR and murine Foxp3 suppress anti-factor VIII immune responses in hemophilia a mice. Cell Immunol. 2020;358:104216. 32987195 10.1016/j.cellimm.2020.104216 PMC7655680

[177] Herzog RW, Kuteyeva V, Saboungi R, Terhorst C, Biswas M. Reprogrammed CD4(+) T cells that Express FoxP3(+) control inhibitory antibody formation in Hemophilia A mice. Front Immunol. 2019;10:274. 30842776 10.3389/fimmu.2019.00274 PMC6391332

[178] Gorgoulis V, Adams PD, Alimonti A, Bennett DC, Bischof O, Bishop C, et al. Cellular Senescence: defining a path Forward. Cell. 2019;179(4):813–27. 31675495 10.1016/j.cell.2019.10.005

[179] Campisi J, Kapahi P, Lithgow GJ, Melov S, Newman JC, Verdin E. From discoveries in ageing research to therapeutics for healthy ageing. Nature. 2019;571(7764):183–92. 31292558 10.1038/s41586-019-1365-2 PMC7205183

[180] Smith HW, Marshall CJ. Regulation of cell signalling by uPAR. Nat Rev Mol Cell Biol. 2010;11(1):23–36. 20027185 10.1038/nrm2821

[181] Amor C, Fernández-Maestre I, Chowdhury S, Ho YJ, Nadella S, Graham C et al. Prophylactic and long-lasting efficacy of senolytic CAR T cells against age-related metabolic dysfunction. Res Square. 2023. 10.1038/s43587-023-00560-5 PMC10950785 38267706

[182] Pereira BI, Devine OP, Vukmanovic-Stejic M, Chambers ES, Subramanian P, Patel N, et al. Senescent cells evade immune clearance via HLA-E-mediated NK and CD8(+) T cell inhibition. Nat Commun. 2019;10(1):2387. 31160572 10.1038/s41467-019-10335-5 PMC6547655

[183] Yang D, Sun B, Li S, Wei W, Liu X, Cui X, et al. NKG2D-CAR T cells eliminate senescent cells in aged mice and nonhuman primates. Sci Transl Med. 2023;15(709):eadd1951. 37585504 10.1126/scitranslmed.add1951

[184] Hu X, Geng Z, Gonelle-Gispert C, Hawthrone WJ, Deng S, Buhler L. International Human Xenotransplantation Inventory: a 10-year follow-up. Transplantation. 2022;106(9):1713–6. 34982756 10.1097/TP.0000000000004016

[185] Parlakpinar H, Gunata M. Transplantation and immunosuppression: a review of novel transplant-related immunosuppressant drugs. Immunopharmacol Immunotoxicol. 2021;43(6):651–65. 34415233 10.1080/08923973.2021.1966033

[186] Xiaojun H, Depei W, Stem Cell Application Group CSoH, Chinese Medical Association. Chinese consensus of allogeneic hematopoietic stem cell transplantation for hematological disease (III) —acute graft-versus-host disease (2020). 2020.

[187] Mo F, Watanabe N, Omdahl KI, Burkhardt PM, Ding X, Hayase E, et al. Engineering T cells to suppress acute GVHD and leukemia relapse after allogeneic hematopoietic stem cell transplantation. Blood. 2023;141(10):1194–208. 36044667 10.1182/blood.2022016052 PMC10023730

[188] Pierini A, Iliopoulou BP, Peiris H, Pérez-Cruz M, Baker J, Hsu K et al. T cells expressing chimeric antigen receptor promote immune tolerance. JCI Insight. 2017;2(20). 10.1172/jci.insight.92865 PMC5846896 29046484

[189] MacDonald KG, Hoeppli RE, Huang Q, Gillies J, Luciani DS, Orban PC, et al. Alloantigen-specific regulatory T cells generated with a chimeric antigen receptor. J Clin Investig. 2016;126(4):1413–24. 26999600 10.1172/JCI82771 PMC4811124

[190] Noyan F, Zimmermann K, Hardtke-Wolenski M, Knoefel A, Schulde E, Geffers R, et al. Prevention of Allograft rejection by Use of Regulatory T cells with an MHC-Specific chimeric Antigen receptor. Am J Transplantation: Official J Am Soc Transplantation Am Soc Transpl Surg. 2017;17(4):917–30. 10.1111/ajt.14175 27997080

[191] Boardman DA, Philippeos C, Fruhwirth GO, Ibrahim MA, Hannen RF, Cooper D et al. Expression of a Chimeric Antigen Receptor Specific for Donor HLA Class I enhances the Potency of Human Regulatory T Cells in preventing human skin transplant rejection. American journal of transplantation: official journal of the American Society of Transplantation and the American Society of Transplant Surgeons. 2017;17(4):931–43. 10.1111/ajt.14185 28027623

[192] Sheldon S, Poulton K. HLA typing and its influence on organ transplantation. (Clifton NJ). 2006;333:157–74. Methods in molecular biology. 10.1385/1-59745-049-9:157 16790851

[193] Patel SA, Spiegel JY, Dahiya S. Second primary Cancer after chimeric Antigen Receptor–T-Cell therapy: a review. JAMA Oncol. 2024. 10.1001/jamaoncol.2024.5412 39666320

[194] Abou-El-Enein M. The fate(s) of CAR T-Cell therapy: navigating the risks of CAR + T-Cell malignancy. Blood cancer Discovery. 2024;5(4):249–57. 38713831 10.1158/2643-3230.BCD-23-0272 PMC11215381

[195] Hamilton MP, Sugio T, Noordenbos T, Shi S, Bulterys PL, Liu CL, et al. Risk of second tumors and T-Cell lymphoma after CAR T-Cell therapy. N Engl J Med. 2024;390(22):2047–60. 38865660 10.1056/NEJMoa2401361 PMC11338600

[196] Sayadmanesh A, Yekehfallah V, Valizadeh A, Abedelahi A, Shafaei H, Shanehbandi D, et al. Strategies for modifying the chimeric antigen receptor (CAR) to improve safety and reduce toxicity in CAR T cell therapy for cancer. Int Immunopharmacol. 2023;125:111093. 37897950 10.1016/j.intimp.2023.111093

[197] Jin G, Liu Y, Wang L, He Z, Zhao X, Ma Y, et al. A single infusion of engineered long-lived and multifunctional T cells confers durable remission of asthma in mice. Nat Immunol. 2024;25(6):1059–72. 38802511 10.1038/s41590-024-01834-9

[198] Benmebarek MR, Karches CH, Cadilha BL, Lesch S, Endres S, Kobold S. Killing Mechanisms of Chimeric Antigen Receptor (CAR) T cells. Int J Mol Sci. 2019;20(6). 10.3390/ijms20061283 PMC6470706 30875739

